# Toward Novel [^18^F]Fluorine-Labeled Radiotracers for the Imaging of α-Synuclein Fibrils

**DOI:** 10.3389/fnagi.2022.830704

**Published:** 2022-04-29

**Authors:** Bright C. Uzuegbunam, Junhao Li, Wojciech Paslawski, Wolfgang Weber, Per Svenningsson, Hans Ågren, Behrooz Hooshyar Yousefi

**Affiliations:** ^1^Department of Nuclear Medicine, Technical University of Munich, Munich, Germany; ^2^Department of Physics and Astronomy, Uppsala University, Uppsala, Sweden; ^3^Department of Clinical Neuroscience, Karolinska Institute, Stockholm, Sweden; ^4^Department of Nuclear Medicine, Philipps University of Marburg, Marburg, Germany

**Keywords:** positron emission tomography (PET), PET tracer development, α-synucleinopathies, α-synuclein aggregates, ruthenium-mediated deoxyfluorination, disarylbisthiazole (DABTA)

## Abstract

The accumulation of α-synuclein aggregates (α-syn) in the human brain is an occurrence common to all α-synucleinopathies. Non-invasive detection of these aggregates in a living brain with a target-specific radiotracer is not yet possible. We have recently discovered that the inclusion of a methylenedioxy group in the structure of diarylbisthiazole (DABTA)-based tracers improves binding affinity and selectivity to α-syn. Subsequently, complementary *in silico* modeling and machine learning (ML) of tracer–protein interactions were employed to predict surface sites and structure–property relations for the binding of the ligands. Based on this observation, we developed a small focused library of DABTAs from which 4-(benzo[d][1,3]dioxol-5-yl)-4′-(3-[^18^F]fluoro-4-methoxyphenyl)-2,2′-bithiazole **[**^18^**F]d**_2_, 6-(4′-(3-[^18^F]fluoro-4-methoxyphenyl)-[2,2′-bithiazol]-4-yl)-[1,3]dioxolo[4,5-b]pyridine **[**^18^**F]d**_4_, 4-(benzo [*d*][1,3]dioxol-5-yl)-4′-(6-[^18^F]fluoropyridin-3-yl)-2,2′-bithiazole **[**^18^**F]d**_6_, and 6-(4′-(6-[^18^F]fluoropyridin-3-yl)-[2,2′-bithiazol]-4-yl)-[1,3]dioxolo[4,5-*b*]pyridine **[**^18^**F]d**_8_ were selected based on their high binding affinity to α-syn and were further evaluated. Binding assay experiments carried out with the non-radioactive versions of the above tracers **d**_2_, **d**_4_, **d**_6_, and **d**_8_ showed high binding affinity of the ligands to α-syn: 1.22, 0.66, 1.21, and 0.10 nM, respectively, as well as excellent selectivity over β-amyloid plaques (Aβ) and microtubular tau aggregates (>200-fold selectivity). To obtain the tracers, their precursors were radiolabeled either *via* an innovative ruthenium-mediated (S_N_Ar) reaction (**[**^18^**F]d**_2_ and **[**^18^**F]d**_4_) or typical S_N_Ar reaction (**[**^18^**F]d**_6_ and **[**^18^**F]d**_8_) with moderate-to-high radiochemical yields (13% – 40%), and high molar activity > 60 GBq/μmol. Biodistribution experiments carried out with the tracers in healthy mice revealed that **[**^18^**F]d**_2_ and **[**^18^**F]d**_4_ showed suboptimal brain pharmacokinetics: 1.58 and 4.63 %ID/g at 5 min post-injection (p.i.), and 1.93 and 3.86 %ID/g at 60 min p.i., respectively. However, **[**^18^**F]d**_6_ and **[**^18^**F]d**_8_ showed improved brain pharmacokinetics: 5.79 and 5.13 %ID/g at 5 min p.i.; 1.75 and 1.07 %ID/g at 60 min p.i.; and 1.04 and 0.58 %ID/g at 120 min p.i., respectively. The brain uptake kinetics of **[**^18^**F]d**_6_ and **[**^18^**F]d**_8_ were confirmed in a dynamic PET study. Both tracers also showed no brain radiometabolites at 20 min p.i. in initial *in vivo* stability experiments carried out in healthy mice. **[**^18^**F]d**_8_ seems very promising based on its binding properties and *in vivo* stability, thus encouraging further validation of its usefulness as a radiotracer for the *in vivo* visualization of α-syn in preclinical and clinical settings. Additionally, *in silico* and ML-predicted values correlated with the experimental binding affinity of the ligands.

## Introduction

In a group of neurodegenerative disorders (NDDs) known as the α-synucleinopathies, the highly soluble presynaptic protein α-synuclein (Uéda et al., 1993; Spillantini et al., 1998; Clayton and George, 1999; Vilar et al., 2008; Uzuegbunam et al., 2020; Korat et al., 2021) is predominantly present as fibrillary insoluble protein aggregates (α-syn) included in Lewy bodies (LB), Lewy neurites (LN), and glial cytoplasmic inclusions (GCI). NDDs characterized by α-syn deposition include Parkinson’s disease (PD), Lewy body dementia (LBD), and multiple system atrophy (MSA) (Tu et al., 1998; Wakabayashi et al., 1998; Spillantini and Goedert, 2000; Burré et al., 2010).

Compelling evidence points at the role of α-synuclein in the pathogenesis of PD. The identification of A53T point mutation in 1996 in familial cases of PD first linked α-synuclein to PD (Polymeropoulos et al., 1997), a finding that was further validated by a later discovery that duplication and triplication of the synuclein-alpha (SNCA) gene is sufficient to cause PD, with an earlier onset of the disease and its severity correlating with higher genetic copies of α-synuclein (Singleton et al., 2003; Chartier-Harlin et al., 2004; Ibáñez et al., 2004).

There is substantial evidence that the accumulation of α-syn precedes the typical clinical symptoms which characterize the synucleinopathies by several years (Riederer and Wuketich, 1976; Fearnley and Lees, 1991; Braak et al., 2003; Ross et al., 2004; Hawkes, 2008). Thus α-syn is an interesting biomarker for the early identification of synucleinopathies and the differential diagnosis of the different types of synucleinopathies.

So far, making a definite diagnosis of the α-synucleinopathies is based on postmortem examination of the brain, especially in sporadic cases without genetic markers (Alafuzoff and Hartikainen, 2017). Even clinical diagnosis which relies on the clinical presentations of the disease has an accuracy of 76%–92% (Bagchi et al., 2013). For this reason, there is an unmet medical need to develop a non-invasive imaging method to detect, quantify, and localize α-syn in the living brain.

Increasing progress has been made so far in the development of selective PET radioligands for the imaging of the biomarkers of other NDDs like Alzheimer’s disease and tauopathies, some of which have gained also regulatory approval such as the tau tracer Flortaucipir (Neurology live, 2021) and the β-amyloid tracers Neuraceq (FDA/CDERB)^1^, Amyvid (FDA/CDERA)^2^, and Vizamyl (FDA)^3^ while some have made to it clinical studies. For the α-synucleinopathies, there is yet to be a radiotracer, which has made it past the preclinical stages (Verdurand et al., 2018; Uzuegbunam et al., 2020; Korat et al., 2021). This could be owing to the following reasons: low binding affinity to α-syn, suboptimal selectivity for α-syn in comparison to Aβ and tau (Ono et al., 2016) with a similar β-sheet core structure (Miranda-Azpiazu et al., 2020). Even when binding affinity and selectivity are suitable, their high lipophilicity (Kaide et al., 2020), *in vivo* instability (Kuebler et al., 2020), might also be limiting factors (Chu et al., 2015).

**FIGURE 1:**
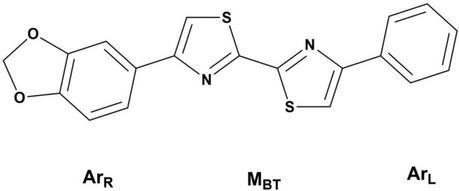
The disarybisthiazole scaffold with a methylenedioxy group.

Not long ago, ligands based on a disarylbisthiazole (DABTA) scaffold were identified and patented (Figure 1) (PubChem, 2022). These ligands showed high binding affinity to α-syn and excellent selectivity over Aβ plaques and tau fibrils in the presence of adequate functional groups. The usage of aryl in the name of this group of ligands, does not preclude the presence of heteroaryl moieties in the ligands. The scaffold for easy description was conveniently divided into the right hetero-/aryl part (Ar_R_), the middle bisthiazole part (M_BT_), and the left hetero/aryl part (Ar_L_) (Figure 1). The DABTA framework, however, has already been introduced by Niu et al. (2020), but as Aβ ligands, which lack functional group(s) unique to the tracers that will be reported in this article.

The DABTAs were designed such that they could be easily labeled with [^18^F]fluorine due to its ideal combination of a 110-min physical half-life (logistically enables transport to satellite PET scanning facilities) in comparison to [^11^C]carbon, whose shorter half-life (∼20 min) limits its usage to centers with on-site cyclotron; a clean decay profile (97% positron emission and 3% electron capture); and a low positron energy (maximum 0.635 MeV), which results in fairly short positron range (maximum 2.4 mm in water) hence high-resolution PET images (∼1 mm) (Shah and Westwell, 2007; Ariza et al., 2015; Jacobson et al., 2015; Edem et al., 2019) and lower patient dose per positron emission (Bailey et al., 2005; van der Born et al., 2017).

To begin with, a series of hetero-/aryl DABTAs with different functional groups initially were synthesized and screened in competition binding assays in which it was discovered that the inclusion of a methylenedioxy moiety (Figure 1) in the structure of the DABTAs significantly improved binding affinity to α-syn and selectivity over Aβ plaque and tau fibrils (PubChem, 2022). In the absence of this methylenedioxy moiety and in the presence of certain functional groups, the DABTAs might lose their high affinity for α-syn with improvement in affinity to the other proteinopathies (PubChem, 2022; Niu et al., 2020). For further development of the DABTAs as α-syn tracers, it made sense to leave the methylenedioxy moiety constant in (Ar_R_) and modify (Ar_L_) and the aromatic ring in (Ar_R_) to further increase binding affinity and selectivity of the tracers, as well as *in vivo* pharmacokinetics of the DABTAs.

## Materials and Methods

### Methods

#### General Synthesis Procedure

##### Chemical Synthesis

Detailed procedures of the syntheses of the DABTAs is described in the Supplementary Material.

**FIGURE 2:**
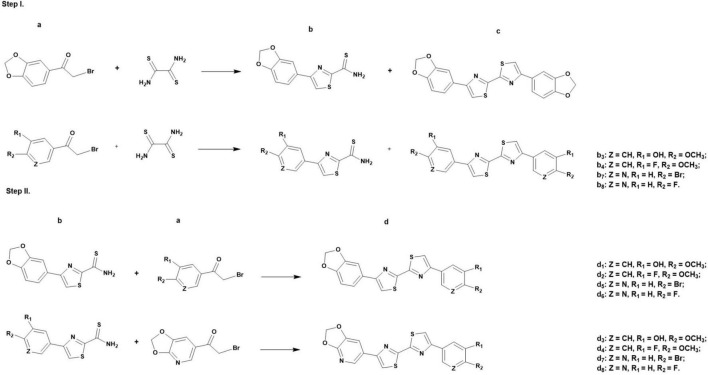
General synthesis of the DABTAs (d_1_-d_8_) in dimethylformamide, where a = aromatic/a heteroaromatic α-bromoketone, b = 4-arylthiazole-2- carbothioamide, c = symmetric side product, and d = asymmetric product.

Generally, the synthesis of the DABTAs follows a stepwise condensation of an aromatic/a heteroaromatic α-bromoketone (labeled as **a** in Figure 2, Step I) with dithiooxamide in anhydrous DMF. After which, the reaction mixture is centrifuged and the supernatant, which contains the desired product (**b**), is decanted from the symmetric side product (**c**) (Figure 2, Step I) and then purified using a C18-reversed phase (RP) column *via* semipreparative HPLC. After the drying of the 4-arylthiazole-2-carbothioamide (**b**), it is coupled with the next aromatic/heteroaromatic α-bromoketone (**a**) (Figure 2, Step II) and the resulting product (**d**) is purified *via* trituration or semipreparative HPLC.

##### Radiosynthesis

###### Radiosynthesis of 4-(benzo[d][1,3]dioxol-5-yl)-4′- (3-[^18^F] fluoro-4-methoxyphenyl)-2,2′-bithiazole ([^18^F]d_2_) and 6-(4′-(3-[^18^F]fluoro-4-methoxyphenyl)-[2,2′-bithiazol]-4-yl)-[1,3] dioxolo[4,5-b]pyridine ([^18^F]d_4_)

**FIGURE 3:**
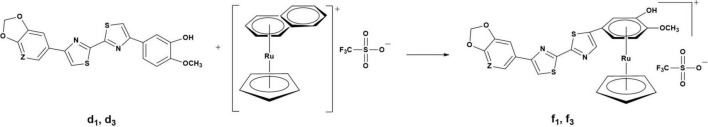
General synthesis of the DABTAâ ruthenium complexes in a mixture of dichloroethane and acetonitrile at 60°C for 24h with (η5-cyclopentadienyl) (η6-naphthalene)ruthenium(+)trifluoromethanesulfonate. Where: Z = N, CH.

Chromafix PS-HCO_3_-^18^F separation cartridge (45.0 mg) (Product No. 731876 from ABX) was pre-conditioned by sequentially pushing an aqueous solution of potassium oxalate (3 mL, 10 mg/mL) and H_2_O (2 mL) through the cartridge at a flow rate of 5 mL/min. Aqueous [^18^F]fluoride solution was loaded in the above preconditioned Chromafix PS-HCO_3_-^18^F cartridge, followed by a wash with anhydrous acetonitrile (1 mL). Air (2 mL) was pushed through the cartridge. The [^18^F]fluoride was eluted from the cartridge with a solution of 7 mg each of the ruthenium complexes of (**f**_1_) and (**f**_3_) (10 mmol) (Figure 3), and 14 mg of 2-chloro-1,3-bis(2,6-diisopropylphenyl)imidazolium chloride (g) (30 mmol, 3.0 equivalent) in methanol (0.5 mL) into a 5 mL borosilicate vial (98% elution efficiency). Methanol was removed by heating at 80°C under a stream of nitrogen (∼5 min). To the vial was added a mixture of ethanol, veratrole, and pivalonitrile (0.45 mL, 1:4:4, v:v:v). The reaction vial containing 0.45 mL of the reaction mixture was sealed with a Teflon-lined cap and was stirred at 130°C for 30 and 35 min for (**f**_1_) and (**f**_3_) respectively (Figure 7) (Beyzavi et al., 2017).

The vial was removed from the heating block, and after 2 min of cooling at room temperature, the reaction mixture was analyzed by RP analytical-HPLC.

The reaction mixture was diluted with 1 mL of 90% aqueous acetonitrile and directly injected in a 2 mL loop for semipreparative HPLC purification (In-line HPLC detectors included a UV detector (Sykam) set at 254 nm and a radioactivity detector (Bioscan Flow-Count fitted with a PIN detector), equipped with a semipreparative column Zorbax Bonus RP, 9.4 × 250 mm, 5.0 μm. **[**^18^**F]d**_2_ and **[**^18^**F]d**_4_ were eluted isocratically with aqueous acetonitrile solution (73 and 63%, respectively) containing 0.01% tetrabutylammonium hydroxide (TBAH) as the ion-pairing agent at retention times (T_R_) 14.0 min (339.5−464.0 MBq) and 15.3 min (225.0−512.1 MBq), respectively. A radiochemical yield (RCY) of 13.7%–15.9% and 12.9%–17.7% for **[**^18^**F]d**_2_ and **[**^18^**F]d**_4_, respectively, were obtained. The > 99% pure fractions obtained from the semipreparative HPLC containing the ^18^F-labeled tracers were diluted with Milli-Q water (1:4.5) and fixed in a Sep-Pak C18 Classic Cartridge, 55–105 μm cartridge (pre-conditioned by passing 2 mL of ethanol through it, and thereafter 10 mL of Milli-Q water). The cartridge was then washed with 4 mL of Milli-Q water. Residual water was removed by pushing nitrogen gas through it. The radiolabeled products were then eluted with 96% ethanol. The ethanol eluate was tested for purity using the reversed-phase (RP) analytical HPLC. The identities of the radiolabeled tracers were confirmed by a spike injection of their cold references **d**_2_ and **d**_4_.

###### Radiosynthesis of 4-(benzo[d]**[1,3]**dioxol-5-yl)-4′-(6-[^18^F] fluoropyridin-3-yl)-2,2′- bithiazole (**[**^18^**F]d**_6_)

**FIGURE 4:**
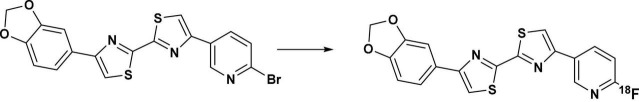
Radiosynthesis of **[**^18^**F]d**_6_ via the precursor **d**_5_, with K_222_ and K_2_CO_3_ at 180°C for 5 min in DMSO.

To obtain **[**^18^**F]d**_6_, aqueous [^18^F]fluoride solution was loaded in a pre-conditioned QMA (Sep-Pak Accell Plus QMA Carbonate Plus Light Cartridge, 130.0 mg sorbent per cartridge, 37–55 μm), which was pre-conditioned by passing 10 mL of Milli-Q water through it. The [^18^F]fluoride was subsequently eluted into a 5 mL borosilicate vial equipped with a magnetic stirrer with 500 μL of aqueous acetonitrile solution containing a mixture of Kryptofix 2.2.2 (7.3 mg, 19.4 μmol) and 6.5 μL of an aqueous solution of 1 M K_2_CO_3_ (6.5 μmol). This was followed by azeotropic drying using 0.5–1 mL anhydrous acetonitrile. After which, 2 mg (4.5 μmol) of the precursor **d**_5_ (Figure 2 [Step II] and Figure 4) was mixed with 0.4 mL DMSO and then added to the above borosilicate vial. The mixture was heated for 5 min at 180°C (Figure 4) then allowed to cool at room temperature. 1 mL of 90% aqueous acetonitrile was added to the reaction mixture, and it subsequently was directly injected in the semipreparative-HPLC and eluted isocratically with 71% aqueous acetonitrile with 0.01% of the ion-pairing reagent TBAH (1.4 N) at a flowrate of 3 mL/min; T_R_ of 14.8 min with 501.0–941.9 MBq (RCY of 18.0%–40.1%) of the tracer obtained. The product fraction was diluted (1:4.5) with Milli-Q water. Further workup followed as described above in section “Radiosynthesis of 4-(benzo[d]**[1,3]** dioxol-5-yl)-4′- (3-[^18^F]fluoro-4-methoxyphenyl)-2,2′-bithiazole (**[**^18^**F]d**_2_) and 6-(4′-(3-[^18^F]fluoro-4-methoxyphenyl)-[2,2′-bithiazol]-4-yl)-**[1,3]**dioxolo[4,5-b]pyridine (**[**^18^**F]d**_4_).” The identity of the **[**^18^**F]d**_6_ was confirmed by a spike injection of its cold reference **d**_6_.

###### Radiosynthesis of 6-(4′-(6-[^18^F]fluoropyridin-3-yl)-[2,2′-bithiazol]-4-yl)-[1,3]dioxolo[4,5-b]pyridine ([^18^F]d_8_)

**FIGURE 5:**
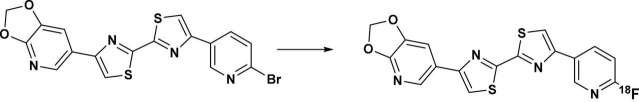
Radiosynthesis of **[**^18^**F]d**_8_ via the precursor **d**_7_, with K_222_ and K_2_CO_3_/KHCO_3_ at 190°C for 6 min in DMSO.

In the case of **[**^18^**F]d**_8_, the [^18^F]fluoride was eluted with a mixture of Kryptofix 2.2.2 (5.1 mg, 13.55 μmol), 4.5 μL of an aqueous solution of 1 M K_2_CO_3_ (4.5 μmol), and 9 μL of an aqueous solution of 1 M KHCO_3_ (9 μmol) in 0.5 mL of acetonitrile. Azeotropic drying followed the same scheme as that of **[**^18^**F]d**_6_. This was followed by heating 2 mg (4.5 μmol) of the precursor **d**_7_ (Figure 5) at 190°C in 0.40 mL DMSO for 6 min in a 5 mL borosilicate vial. The workup follows a similar scheme as **[**^18^**F]d**_6_. Semipreparative HPLC purification was carried out with 62% aqueous acetonitrile with 0.01% of the ion-pairing reagent TBAH (1.4 N) at a flow rate of 3 mL/min; T_R_ of 11.9 min with 301.4–750.6 MBq (RCY of 13.5–25.0%) of the tracer obtained. The product fraction was diluted (1:4.5) with Milli-Q water. Further workup followed the same procedure as already described above in section “Radiosynthesis of 4-(benzo[d]**[1,3]**dioxol-5-yl)-4′- (3-[^18^F]fluoro-4-methoxyphenyl)-2,2′-bithiazole (**[**^18^**F]d**_2_) and 6-(4′-(3-[^18^F]fluoro-4-methoxyphenyl)-[2,2′-bithiazol]-4-yl)-**[1,3]**dioxolo[4,5-b]pyridine (**[**^18^**F]d**_4_).” The identity of the **[**^18^**F]d**_8_ was confirmed by a spike injection of its cold reference **d**_6_

#### *In vitro* Experiments

##### Log D Determination

To a solution of **[**^18^**F]d**_2_, **[**^18^**F]d**_4_, **[**^18^**F]d**_6_, and **[**^18^**F]d**_8_ (0.5–1.0 MBq) in 0.5 mL octanol, 0.5 mL PBS (pH 7.4) was added in a 1.5 mL Eppendorf vial. The vials were vigorously vortexed for 10 min. To achieve efficient phase separation, the vials were centrifuged for 10 min at 13,000 rpm in a Heraeus Biofuge 13 centrifuge. Aliquots (100 μL) of the aqueous and the octanol phase were collected carefully and the amount radioactivity in the respective samples was quantified using a γ-counter. The log octanol/PBS values were calculated from the means of *n* = 3 separate determinations.

##### *In vitro* Plasma Stability Experiments

They were performed with Seronorm Human plasma (Invicon Gmbh, Munich, Germany). An aliquot of the radiotracers (5-10 MBq) of **[**^18^**F]d**_2_, **[**^18^**F]d**_4_, **[**^18^**F]d**_6_, and **[**^18^**F]d**_8_ was added to human plasma (300 μL) and incubated at 37 C. After 30-, 60-, 90-, and 120 min aliquots were taken, and plasma protein was precipitated with 250 μL acetonitrile. The resulting mixtures were centrifuged for 10 min at 13,000 rpm in a Heraeus Biofuge 13 centrifuge and the supernatants were analyzed by radio-HPLC (*n* = 3).

##### *In vitro* Plasma Protein Binding Experiments

To estimate the free fraction (*fp*) of the radioligands in the plasma, an ultrafiltration method was used. Approximately 1 MBq of **[**^18^**F]d**_2_, **[**^18^**F]d**_4_, **[**^18^**F]d**_6_, and **[**^18^**F]d**_8_ were added to 450 μL of Seronorm Human plasma (Invicon GmbH, Munich, Germany), and the mixture was incubated for 10 min. After incubation, 150 μL of plasma was transferred into 3 ultrafiltration tubes (Centrifree YM-30; cutoff 30,000 MW; Millipore) and centrifuged at 8,000 rpm for 10 min. Equal aliquots of the ultrafiltrate (*C*_free_), and of the plasma (*C*_total_) were counted for their radioactivity with a γ-counter. The *fp* of the radioligands was calculated as *f_*p*_* = *C_free_/C_total_* (*n* = 3).

##### Competition Binding Assays

###### Competition Binding Assays for the α-Syn Fibrils

A fixed concentration of [^3^H]DCVJ (10 nM) a pan-amyloid ligand (K_d_ for α-syn 4.4 nM, Aβ 8.9 nM, tau 15.6 nM), and α-syn (125 nM) prepared as described previously (Paslawski et al., 2016, 2019) were used with different concentrations of **d**_2_, **d**_4_, **d**_6_, and **d**_8_ from 0.01 to 100 nM. The competitor reaction was diluted with 20 mM Tris-HCl, pH 7.4 to a final volume of 200 μL per well. After incubation for 2 h at 37°C, the binding mixtures were filtered through a Perkin Elmer GF/B glass filter *via* a TOMTEC cell harvester and immediately washed three times with 1 mL of deionized water. Filters containing the bound ligands were dried and mixed with 3 mL of Betaplate scint solution (PerkinElmer Life Sciences) and incubated for 2 h before counting in **a Wallac 1450 MicroBeta TriLux Liquid Scintillation Counter** (PerkinElmer Life Sciences). For the determination of the inhibition constants, data were analyzed using GraphPad Prism software (version 7.0) to obtain IC_50_ values by fitting the data to the equation Y = bottom + (top - bottom)/(1 + 10(x - log IC_50_). K_i_ values were calculated from IC_50_ values using the equation K_i_ = IC_50_/(1 + [radioligand]/K_d_).

###### Competition Binding Assays for Aβ_1–42_, and Tau Fibrils

Fixed concentrations (125 nM) of Aβ_1–42_, tau aggregates, and [^3^H]DCVJ (10 and 20 nM, respectively) were used with different concentrations of cold **d**_2_, **d**_4_, **d**_6_, and **d**_8_ from 1 to 1,000 nM. The experiment was performed in quadruplicates for each concentration. Further procedures followed the same already described in section “Competition Binding Assays for α-Syn Fibrils*.”*

Details about the preparation of the fibrils can be found in the Supplementary Material.

#### *Ex vivo* and *in vivo* Experiments

##### Biodistribution Experiments

The ethanol solutions containing the tracers **[**^18^**F]d**_2_, **[**^18^**F]d**_4_, **[**^18^**F]d**_6_, and **[**^18^**F]d**_8_ were diluted with DMSO, Tween-20, and saline added consecutively to achieve a final concentration of 2.4, 2.4, and 0.4% of ethanol, DMSO, and Tween-20, respectively. The pH was adjusted with 0.05 N aqueous sodium hydroxide solution. 1.5–2.5 MBq of **[**^18^**F]d**_2_, **[**^18^**F]d**_4_, **[**^18^**F]d**_6_, and **[**^18^**F]d**_8_ prepared in the above solvent mixture were administered to the mice *via* the caudal vein under isoflurane anesthesia. The mice were sacrificed at 5 and 60 min p.i. The following organs/tissues were extracted: blood, heart, lung, liver, stomach, pancreas, colon, small intestine, kidney, muscle, bone, tail, and the brain. The radioactivity in the pre-weighed tissue samples was determined using a γ-counter. Data are expressed as a percent of the injected dose per gram tissue (% ID/g; mean ± sd, *n* = 3).

##### *In vivo* PET-MRI Dynamic Scan With Biodistribution

For PET imaging **[**^18^**F]d**_6_ and **[**^18^**F]d**_8_ were prepared as described in section “Biodistribution Experiments” and ∼11 MBq was injected *via* the caudal vein in the mice (*n* = 3 for each tracer). PET was recorded on nanoScan PET/MRI (Mediso Medical Imaging System) under isoflurane anesthesia. PET was continuously recorded in list mode for 120 min and consisted of 36 frames (1 × 60 s; 30 × 10 s; 1 × 600 s; 1 × 900 s; and 3 × 1800 s) while anesthesia was maintained. Data were reconstructed using Nucline nanoScan 3.00.021.0000 software, employing a three-dimensional ordered subset expectation maximum (OSEM3D) algorithm without a scatter and attenuation correction. Tissue time–activity curves (TACs) were obtained by projecting the defined ROI onto all frames of the dynamic PET scans. At 2 h after the PET dynamic scan, the mice were promptly euthanized, the rest of the protocol followed as already described in section “Biodistribution Experiments.”

Magnetic resonance imaging scan is a Fast spin Echo 2D (FSE2D) scan, T2-weighted with Coronal Orientation, 23 slices with a slice thickness of 1.0 mm and a 0.2 mm slice gap. Frequency resolution was 0.391 mm and phase resolution 0.198 mm. The number of excitations was 2, number of repetition time TR shots 1, TR 3,000 ms, and echo time (TE) 60.6 ms. Frequency field-of-view (FOV) 100 mm, phase FOV 38 mm, frequency encoding 256, phase encoding 192, echo train length 8, and echo spacing 7.58 ms.

##### Metabolite Experiments

Tracers **[**^18^**F]d**_6_ and **[**^18^**F]d**_8_ were prepared as already described above in section “Biodistribution Experiments.” About 80–120 MBq of the tracers were injected *via* the caudal vein in the mice (*n* = 3 per tracer). At 20 min p.i., the animals were euthanized, and the needed tissues were extracted and placed on dry ice. To separate plasma from the blood cells, the blood samples were first centrifuged 13,000 rpm for 10 min, then acetonitrile acidified with 1% trifluoroacetic acid (TFA) (10 min, 4°C) was added to the centrifugate to precipitate the plasma proteins, followed by another centrifugation as already described, and ultrafiltration using ultrafiltration tubes (Centrifree YM-30; cutoff 30,000 MW; Millipore). A 10 μL of 1 M DMSO solution of the cold references was added to the centrifugate which was subsequently analyzed by RP-HPLC. The brain was frozen in liquid nitrogen and homogenized with a ball mill. A mixture of acetonitrile and DMSO (5:1) with 1% TFA containing the cold references of the tracers was added to the homogenate and mixed. The suspension was centrifuged. The supernatant was then filtered using ultrafiltration tubes as already described, the resulting filtrate was subsequently analyzed by RP-HPLC.

## Results and Discussion

### Chemical Synthesis

The synthesis of the DABTAs follows a modified Hantzsch method of thiazole synthesis (Wu, 2015), steps which have already been shown in Figure 2. Since (**a**) (Figure 2, Step I) is the limiting reagent, a 1.5 molar equivalent (mol. equiv.) of dithiooxamide is mostly used. Although not conclusively researched, this seems to be adequate as more dithiooxamide (>1.5 mol. equiv.) complicates the purification of the (**b**) that results in re-purification, which leads to further loss of (**b**) and generally is uneconomical.

The first step of the synthesis can also be carried out in ethanol, although it takes place at a slower rate. However, the main reason why it was avoided was due to the solubility of the products (**b**) and (**c**) formed in the first step of the reaction. Product (**b**) is in most cases moderately soluble in ethanol and co-precipitates at room temperature with the usually insoluble side-product (**c**), which complicates semipreparative purification after re-dissolution in a more suitable solvent.

**FIGURE 6:**
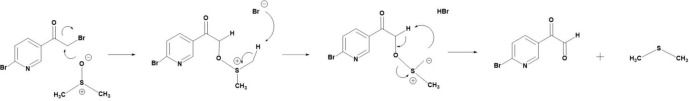
The proposed reaction mechanism of the formation of a glyoxal and dimethyl sulfide from an α-bromoketone and DMSO (Kornblum et al., 1957; Imperial College London, 2021).

Although also a strong organic solvent capable of keeping (**b**) in solution, DMSO was routinely avoided because of the side-reaction(s) (Figure 6) that ensue(s) upon the addition of the α-bromoketones (**a**) to a solution of the nucleophilic DMSO (Kornblum et al., 1957; Smith and Winstein, 1958; Imperial College London, 2021) with pK_a_ 35, in which the acidity of the α-carbon in (**a**) is further improved hence facilitating a nucleophilic attack on the α-carbon. The above side-reaction (Figure 6) is also possible in DMF (Kornblum and Blackwood, 1956) but occurs at a much slower rate (Imperial College London, 2021).

Product (**b**) (Figure 2, Step I) is preferentially purified using the semipreparative HPLC to achieve >99% purity, since the final products (**d**) are mostly hardly soluble or almost insoluble in the commonly used strong organic solvents, such as DMSO, DMF, tetrahydrofuran (THF), and dimethylacetamide (DMA), hence are not amenable to purification using the semipreparative HPLC. This enables easier and cleaner chemistry by getting rid of almost all the dithiooxamide and aromatic/heteroaromatic α-bromoketone (**a**), which may engage in side-reactions in Step II (Figure 2) of the reaction and give rise to products that encumber the purification of (**d**). This is also why a one-pot synthesis of both (**b**) and (**d**) is avoided.

Hence after the Step I of the synthesis (**b**) is promptly separated from (**c**) *via* centrifugation and the supernatant is decanted, and subsequently purified. Purification is accomplished using 20–40% aqueous methanol solution (solvent A). An aqueous tetrahydrofuran solution can be used to this end, but it is mostly preferred as a modifier in the aqueous methanol solution. These solvent mixtures ensure that there is no precipitation in the column or the tubes during purification.

Obtaining product (**b**) in high purity, allows the product (**d**) to be easily purified *via* trituration using methanol, acetone. In which the unreacted (**a**) and (**b**) are soluble (Figure 2, Step II), and (**d**) is poorly soluble. Albeit uneconomical and time-consuming, the guaiacol ligands (**d**_1_ and **d**_3_) can also be purified using the HPLC, with the **d**_1_ being more amenable to this than **d**_3_. A feature common to all the DABTAs, where the addition of a heterocycle counterintuitively reduces solubility in the above-mentioned organic solvents, but at the same time reduces lipophilicity.

### Radiochemical Synthesis

#### Ruthenium-Mediated S_N_Ar Deoxyradiofluorination

**FIGURE 7:**
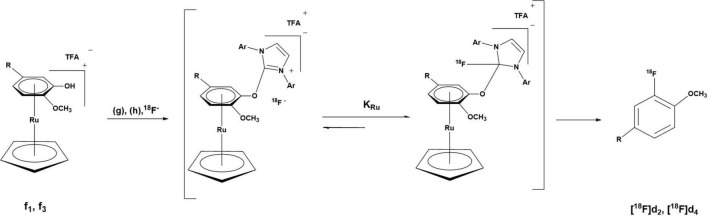
Deoxyradiofluorination of the DABTA-ruthenium complexes at 130°C for 30-35 min, with g = 2-Chloro-1,3-bis(2,6-diisopropylphenyl)imidazolium chloride, h = ethanol: pivalonitrile: veratrole (1:4:4), TFA = trifluoroacetic acid (counteranion obtained as a result of semipreparative purification with TFA-acidified solvents). For “R,” see Figure 3.

The radiofluorination of the precursors (**d**_1_ and **d**_3_) of **[**^18^**F]d**_2_ and **[**^18^**F]d**_4_ was carried out using their ruthenium complexes (**f**_1_ and **f**_3_) *via* a ruthenium-mediated deoxyfluorination of phenols (Figure 7). Typical nucleophilic aromatic substitution reactions (S_N_Ar) require electron withdrawing groups (EWG) which dramatically increases the rate of reaction depending on their position relative to the leaving group (LG). In this case, not only do the precursors have no EWG but the LG, the hydroxy group (-OH), is not good LG. Hence, to facilitate the incorporation of [^18^F]F^–^ in the ring, the ring has to be made sufficiently electron deficient. This was made possible *via* η^6^-haptic coordination of the guaiacol ring to a ruthenium complex (RuCp) (Beyzavi et al., 2017; Neumann and Ritter, 2017).

To this end, **d**_1_ and **d**_3_ were reacted (Figure 3) with (η^5^-cyclopentadienyl)(η^6^-naphthalene)ruthenium(+)trifluorometh- anesulfonate in the low coordinating solvent dichloroethane (DCE) in the presence of 10% v/v of the catalyst acetonitrile (MeCN). Acetonitrile plays the role of an auxiliary ligand and further facilitates the η^6^ → η^4^ ring slippage and eventual removal of the naphthalene ring (Bennett et al., 1998; Pigge and Coniglio, 2001; Perekalin et al., 2012).

The binding of the guaiacol moiety (Figure 3) to the metal center depletes the electron density in the ring, which makes it more susceptible to nucleophilic attack (Figures 3, 7) (Konovalov et al., 2015; Beyzavi et al., 2017; Neumann and Ritter, 2017). The η^6^-coordination of ruthenium to the benzene ring can be likened to that of nitro groups in the ortho and para positions as in picric acid (Konovalov et al., 2015).

The introduction of the nucleophilic [^18^F]F^–^ in the guaiacol ring in **d**_1_ and **d**_3_ follows the traditional S_N_Ar mechanism, not the previously thought concerted S_N_Ar believed to be the case for such electron-rich phenols (Fujimoto et al., 2014). This is due to the stabilization of the Meisenheimer complex by the RuCp fragment that the former is favored over the latter. *Via* a condensation of the deoxyfluorination agent *N*′-1,3-bis(2,6-diisopropylphenyl)chloroimidazolium chloride (g) (Figure 7) with the -OH group in the guaiacol ring a uronium fluoride intermediate is formed, that is in equilibrium with a tetrahedral intermediate which bears a non-basic neutral organofluoride (Figure 7). At this juncture, the positively charged RuCp fragment plays an important role in increasing the coulombic attraction between the guaiacol–reagent complex and the [^18^F]fluoride, thereby increasing the concentration of the tetrahedral intermediate in solution which rearranges to afford the 3-[^18^F]fluoroanisole products (Figure 7) (Beyzavi et al., 2017; Neumann and Ritter, 2017). This is preceded by the detachment of the *in situ* formed LG and the decomplexation of the RuCp facilitated by the solvent (reaction medium), which comprises veratrole, pivalonitrile, and ethanol (4:4:1 v/v) (Jeannin, 1993; Mori and Mochida, 2013; Fujimoto et al., 2014; Beyzavi et al., 2017), a high temperature of 130°C and a reaction duration of 30 min for **f**_1_ and 35 min for **f**_3_.

It is necessary that the reaction duration is as long as was the case or else the 3-[^18^F]fluoroanisole products will remain complexed to the RuCp fragment. Nevertheless, the loss of radiochemical yield (RCY) *via* radioactive decay is compensated for, since radiosynthesis takes place without azeotropic drying as was the case for **[**^18^**F]d**_6_ and **[**^18^**F]d**_8_. Another technical limitation is the requirement of relatively higher quantities of the reagents 7 mg of **f**_1_ and **f**_3_, (Figure 3) and 14 mg of (g) (Figure 7), which complicates the purification of the tracers. Moreover, one of the challenges for the GMP production and translational research of such radiosynthesis based on using RuCp is the analysis of remaining ruthenium in product and the radiopharmacy sector historically has been reluctant to use transition metal catalysts due to toxicity concerns.

Nevertheless, the radiosynthesis afforded up to 13.7%–15.9% and 12.9%–17.7% of **[**^18^**F]d**_2_ and **[**^18^**F]d**_4_, respectively. Under optimal conditions, RCYs of both tracers are expected to be up to 20–22% since some of the tracers get retained in the semipreparative HPLC column. The molar activity was determined to be up to be 64.7–108.0 GBq/μmol for **[**^18^**F]d**_2_ and 104.0–175.6 GBq/μmol for **[**^18^**F]d**_4_.

#### Direct Radiofluorination

The radiolabeling of the precursor of **[**^18^**F]d**_6_ was accomplished *via* an S_N_Ar reaction (Figure 4) using the phase-transfer catalyst Kryptofix 2.2.2. (K_222_), and the inorganic basic salt potassium carbonate (K_2_CO_3_) in anhydrous DMSO to an isolated RCY of 18.0%–40.1% and molar activity 47.5–92.4 GBq/μmol.

The radiosynthesis of **[**^18^**F]d**_6_ was easily carried out and gave moderate to high yields at both 3 min (14 – 17%) and 5 min (29 – 40%) in the same condition. Even up to 4% RCY in harsher conditions with 3.3 mol. equiv. of K_2_CO_3_ and 3.7 mol. equiv. of the K_222_. The latter conditions did not translate at all for the radiolabeling of d_7_ (Figure 5) since no [^18^F]d_8_ was formed. Hence further optimization was attempted in different conditions (Tables 1–3 and Supplementary Material) to obtain **[**^18^**F]d**_8_ in higher RCY. Higher RCY was only possible for d_7_ at a milder basic pH, relatively higher temperature, and longer duration.

**TABLE 1:** MM/GBSA free energies (kcal/mol) for DABTAs and DCV).

Compounds	Ki (nM)	Site-1	Site-2	Site-3	Site-4
d_2_	1.22	−51.3	–^a^	−43.7	–61.6
d_4_	0.66	−48.9	–	−35.8	–66.9
d_6_	1.21	−47.2	–	−38.8	–54.0
d_8_	0.10	−43.5	–	−44.2	–
DCVJ	4.42^b^	−28.9	−36.8	−29.8	−24.8

*^a^No favorable binding poses (docking score < **−**5 kcal/mol) in blind docking.*

*^b^K_d_ value (nM).*

An isolated RCY of up to 25% is possible, with a molar activity of 40–104 GBq/μmol following the procedure described in the section *“Radiosynthesis of 6-(4’-(6-[^18^F]Fluoropyridin-3-yl)-[2,2’-Bithiazol]-4-yl)-[1,3]Dioxolo[4,5-b]Pyridine (**[**^18^**F]*****d***_8_)”*, Figure 5.

### *In silico* Modeling of Disarylbisthiazoles Binding to α-Syn

**FIGURE 8:**
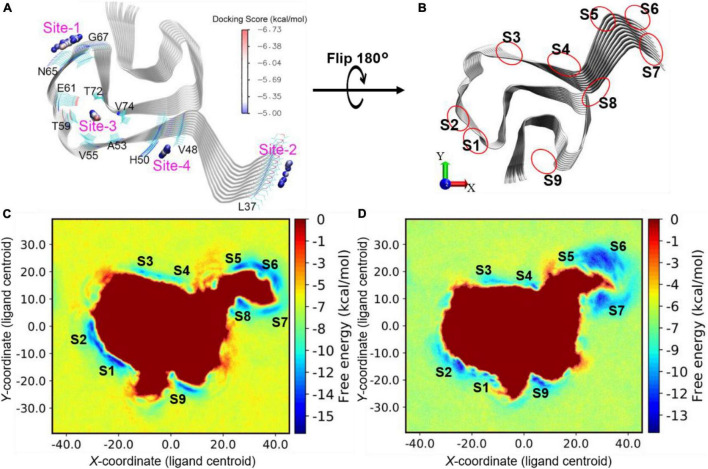
**(A)** Blind docking identified sites (labeled from “Site-1” - “Site-4”) with favorable docking scores (<–5 kcal/mol) for DABTAs and DCVJ. The geometrical centers of the docked ligands are depicted as small spheres and colored by the range of docking scores. **(B)** Illustration of sites identified by metadynamics simulations. **(C)** Free energy profile for d_4_ binding onto the surface sites of α-syn. **(D)** Free energy profile for d_8_ binding onto the surface sites of α-syn. The free energy local minima are named with “S” combined with identity numbers.

To further understand the binding mechanisms of the prospective DABTAs to α-syn, multiple *in silico* techniques were implemented. This enabled to study the interactions between DABTAs and α-syn at an atomic level *via* molecular docking, MM/GBSA (molecular mechanics, the generalized Born model, and solvent accessibility) free energy calculations, and metadynamics simulations (For more details about the modeling setups, see sections 5.2–5.4 in Supplementary Material). From multiple-center glide blind dockings, four potential binding sites for **d**_2_, **d**_4_, **d**_6_, **d**_8_, and DCVJ were identified: Sites 1–4 (Figure 8A). Site-2 stood out in this study in that only the docking of DCVJ to it exhibited favorable scores, which were defined to be lower than −5 kcal/mol. Additionally, it is notable that Site-2 is close to the flexible N-terminal, which might expand when the N-terminal residues disassemble lowering thereby the binding affinity of the ligands to α-syn. Site-3 is an interior site positioned deeper in the fibril, and as revealed by the docking scores, the DABTAs bound more favorably to this site than to the other sites. The MM/GBSA free energies for the DABTAs binding to Sites-1, -3, and -4 were comparatively lower than that of DCVJ (Table 1), indicating that the DABTAs are better tracers for α-syn than DCVJ. Interestingly, for MM/GBSA, in which the flexibility of the protein residues within 5 Å of the ligand was accounted for in the simulations, free energies of binding for ligands **d**_2_, **d**_4_, and **d**_6_ to Site-3 showed less-favorable binding. This indicates that the ligands were most probably bound to the surface sites than to the interior sites. Site-4, markedly, displayed a high binding affinity for **d**_2_, **d**_4_, and **d**_6_ over the other surface sites (Table 1), but it failed to generate a favorable binding pose for **d**_8_. This was reflected by the best-observed docking score for d_8_ at Site-4 to be −3.8 kcal/mol – the corresponding MM/GBSA free energy for the same site was −36.8 kcal/mol. The reason for this could be attributed to the exclusion of protein flexibility and solvent effect in the dockings. For the DABTAs, when qualifying the differences between the MM/GBSA free energies and experimental binding affinity we note that the deviations in K_i_ are about 1.0 kcal/mol.

**FIGURE 9:**
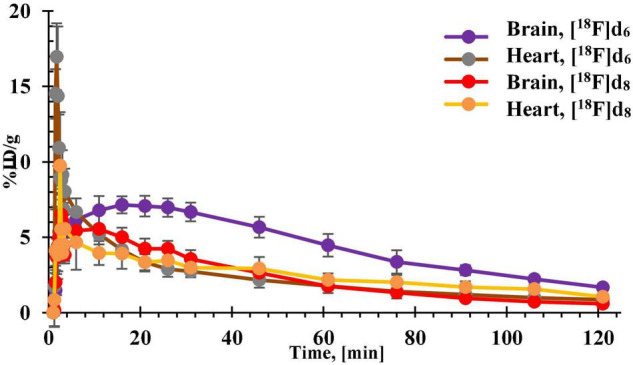
Time-activity curves (TACs) of [^18^F]d_6_ and [^18^F]d_8_ obtained from the brain and heart tissues of healthy C57BL/6J mice.

In the metadynamics simulations, the conformational sampling of the ligand binding and unbinding to the surface of α-syn was accelerated by adding a biased potential to the movement of ligand along the *x-* and *y-*axes. The free energy of the system was then directly correlated to the position of the ligand and the nature of the protein–ligand binding. The more favorable the binding site is, the more negative the free energy becomes. The ligands **d**_4_ and **d**_8_ showed similar binding-free energy profiles in the metadynamics simulations at a gross level (Figure 8), which is reflected by their K_i_ (Table 3) (0.66 and 0.10 nM), which are close to one another. Furthermore, in the free energy profiles of **d**_4_ and **d**_8_, more stable binding sites were identified, the sites S1–S9 (Figure 8). S1 and S4 are the closest sites to the identified docking sites Site-1 and Site-4, respectively. The sites S5, S6, and S7 are located at the N-terminal residues and fused as a large area of local minimum, which results in unfavorable binding. This is likely caused by the large flexibility of the N-terminal residues that were disassembled into fuzzy-like conformations during the metadynamics simulations (Figure 9, SI). In contrast, the C-terminal residues are relatively more rigidly assembled, stabilizing the adjacent sites S1 and S9 (not expanding into a large area, which increases the uncertainty of the position of the binding sites and decreases the binding affinity). The ligand **d**_8_ was found to have strongly favorable binding at sites S2, S4, and S9, while **d**_4_ showed strong binding at the sites S1, S2, and S9 (Figures 8C,D). Remarkably, in the docking and MM/GBSA calculations, Site-4 (S4) is the best binding site for **d**_4_. However, when the solvent effect and protein dynamics were explicitly included, this site became less favorable for the binding of **d**_4_ (see the high-energy local minimum, Figure 8C). Based on the above observations it could be said S2 and S9 are the most favorable surface sites for the binding of the DABTAs (**d**_4_ and **d**_8_) to α-syn.

### *In vitro*, *ex vivo*, and *in vivo* Evaluation of the Radioligands

All the tracers showed no noticeable defluorination or breakdown of any kind after incubation in human plasma at 30, 60, 90, and 120 min as expected.

As was also expected, the exchange of the benzodioxole ring in **[**^18^**F]d**_2_ and **[**^18^**F]d**_6_ with a 2,3-methylenedioxypyridine ring slightly reduced their log D by approximately 3.2 and 7.1% in **[**^18^**F]d**_4_ and **[**^18^**F]d**_8_, respectively (Table 2). This was also reflected in their free plasma fractions (*fp*) which were higher than that of their benzodioxole counterparts (**[**^18^**F]d**_2_ and **[**^18^**F]d**_6_): **[**^18^**F]d**_8_ displayed an *fp* about 6 times higher than that of **[**^18^**F]d**_6_; **[**^18^**F]d**_4_ with an *fp* nearly 2 times higher than **[**^18^**F]d**_2_. Surprisingly, based on their log D, the lowest *fp* was observed in **[**^18^**F]d**_6_.

**TABLE 2:** Some physicochemical properties of the DABTAs.

DABTA	tPSA* [Å^2^]	log D	*fp* [%]
[^18^F]d_2_	52.4	3.1 ± 0.09	0.25 ± 0.01
[^18^F]d_4_	64.8	3.0 ± 0.04	0.45 ± 0.03
[^18^F]d_6_	55.9	2.8 ± 0.09	0.14 ± 0.02
[^18^F]d_8_	67.9	2.6 ± 0.09	0.85 ± 0.02

*tPSA, topological surface area.

**FIGURE 10:**
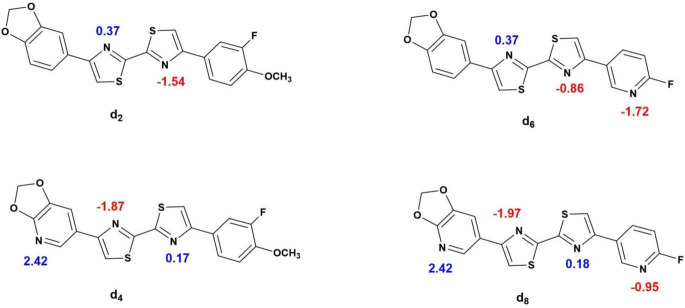
pK_a_ of the ionizable atoms in the d_2_, d_4_, d_6_, and d_8_ (Chemicalize platform).

Their relative binding to the plasma proteins can be partly explained by their relative affinity to the alkalotic plasma albumin, which makes up a majority of the blood plasma proteins (Croom, 2012). Based on the acid dissociation constants (pK_a_) (Chemicalize platform) (Figure 10) of the most basic atoms in the ligands: 0.37 for **[**^18^**F]d**_2_ and **[**^18^**F]d**_6_ and 2.42 for **[**^18^**F]d**_4_ and **[**^18^**F]d**_8_, respectively, it is reasonable that the most acidic in this series displayed the lowest *fp*, nevertheless the *fp* of **[**^18^**F]d**_6_ is still at odds with its log D, when compared to **[**^18^**F]d**_2_. This could also be partly explained by the presence of the methoxy group in the 3-[^18^F]fluoroanisole ligand **[**^18^**F]d**_2_, which although slightly deactivated by the fluorine atom ortho to it, the oxygen atom was still able to form hydrogen bonds with the aqueous medium.

Lacking a methoxy group, **[**^18^**F]d**_6_ relies only on its three heterocyclic nitrogen atoms for hydrogen bonding, two of which are also present in **[**^18^**F]d**_2_. The additional third which is present in the pyridine ring has its basicity diminished significantly by the fluorine atom ortho to it (pk_a_ −1.72) in comparison to pyridine itself (pk_a_ 4.22) (Chemicalize platform) (Chemicalize, 2021) which reduces its ability to form hydrogen bonds (Timperley et al., 2005; Han and Tao, 2006; Chopra et al., 2019; Ogruc Ildiz and Fausto, 2020). **[**^18^**F]d**_8_ has the advantage of having the lowest lipophilicity among the 4 ligands, in addition to a higher topological polar surface area (tPSA), which allows it to interact better with its aqueous environment, since hydrophobic factors in addition to steric and electronic factors all contribute to ligand binding (Smith et al., 2001).

The binding affinity of **[**^18^**F]d**_2_, **[**^18^**F]d**_4_, **[**^18^**F]d**_6_, and **[**^18^**F]d**_8_ to human recombinant α-syn, Aβ, and tau fibrils was assayed in a competition binding assay with non-radioactive ligands **d**_2_, **d**_4_, **d**_6_, and **d**_8_ against tritiated 9-(2,2-dicyanovinyl)julolidine (DCVJ) (Figure 3 and Supplementary Material). DCVJ is a fluorescent probe that binds to α-syn fibrils (Kuang et al., 2019) and the other fibrils with high affinity (K_d_ for α-syn 4.42 nM, Aβ 8.9 nM, tau 15.6 nM). Although being a pan tracer makes DCVJ a less suitable tracer for the *in vivo* imaging of any of the protein aggregates, it makes it, however, a useful tool for evaluating the usefulness of other ligands as tracers for various proteinopathies in competitive binding experiments, as well as for scanning the fibril-binding sites in a set of *in silico* blind dockings.

The DABTAs **d**_2_, **d**_4_, **d**_6_, and **d**_8_ showed a higher affinity for α-syn than DCVJ both in the *in silico* (°K_i_) and experimental (K_i_) studies (Table 3). This is because as seen in Figure 10 and Supplementary Material, the linear scaffold of the DABTAs favors a strong van der Waals interaction with the α-syn backbone, while the more compact structure of the DCVJ molecule prevents such extensive interaction thereby lowering its affinity to α-syn.

**TABLE 3:** The calculated (°K_i_) and experimental (K_i_) binding affinity of the DABTAs to α-syn, Aβ, and tau fibrils with some physicochemical properties.

DABTA	CLogP	°K_i_ [nM]			K_i_ [nM]			Selectivity, (°K_i_)		Selectivity, (K_i_)	
		α-syn	Aβ_1–42_	Tau	α-syn	Aβ_1–42_	Tau	(Aβ/α-syn)	(Tau/α-syn)	(Aβ/α-syn)	(Tau/α-syn)
d_2_	5.5	3.99	382.0	916	1.22	241.7	>1000	96	230	198	>1000
d_4_	4.9	2.17	353.0	916	0.66	275.3	>1000	163	423	417	>1000
d_6_	4.2	3.96	343.0	917	1.21	237.2	966	87	232	196	798
d_8_	3.5	0.33	427.0	1047	0.10	386.3	>1000	1295	3171	3863	>1000

*ClogP, calculated log P (Chemdraw professional).*

*°K_i_:Values in parenthesis are calculated from our in-house machine learning model (section 5 in Supplementary Material).*

As previously mentioned, a series of initial binding assays (Wester and Yousefi, 2020) revealed that the methylenedioxy group is essential to the binding affinity of the DABTAs to our target and selectivity over the other Aβ and tau.

In addition to this, other trends were additionally observed. The bisthiazole scaffold (MB_T_) (Figure 1) also plays a role in the binding affinity of the DABTAs to α-syn, if not selectivity too. However, this depends on the nature of Ar_L_ and Ar_R_, the more electron-withdrawing they are, the higher the affinity to α-syn (Figure 7, Table 3, and Supplementary Material). The role of at least a hydrogen bond (H-bond) acceptor on both peripheries (Ar_L_ and Ar_R_) (Figure 1) was also seen in the 3-fluoroanisole DABTAs, as well as in **d**_8_. H-bond donors, on the other hand, do not necessarily reduce affinity, rather they diminish selectivity over Aβ and tau (Wester and Yousefi, 2020). This could be attributed to the energy costs of desolvation, since a donated H-bond requires nearly twice the energy of an accepted one to be broken (Polêto et al., 2018). Lastly, a higher predisposition to the formation of π–π interactions upon the replacement of a methylidene carbon in the ring with a nitrogen atom, which ultimately improves affinity to α-syn (Huber et al., 2014; Khavasi et al., 2015)] was seen in the 6-fluoropyridine DABTAs and **d**_4_.

The K_i_ of **d**_2_ was determined to be 1.22 nM to α-syn fibrils, with about 200-fold and 1,000-fold (Table 3) selectivity over Aβ and tau, respectively. The replacement of the benzodioxole-moiety with a 2,3-methylenedioxypyridine moiety led to not only an increase in binding affinity to the target 0.66 nM but also to a slightly decreased affinity to Aβ fibrils (1.1-fold) in comparison to d_2_ (Figures 6, 7, Table 3, and Supplementary Material). Due to the additional Lewis base in the 2,3-methylenepyridine ring, there might have been an increased tendency to H-bonding and formation of π–π interactions. Improvement in selectivity over Aβ could be explained by the overall decrease in lipophilicity, which must have led to decreased non-specific binding to the β-sheet present in these protein aggregates (Mori and Mochida, 2013) due to decreased hydrophobic interactions.

**FIGURE 11:**
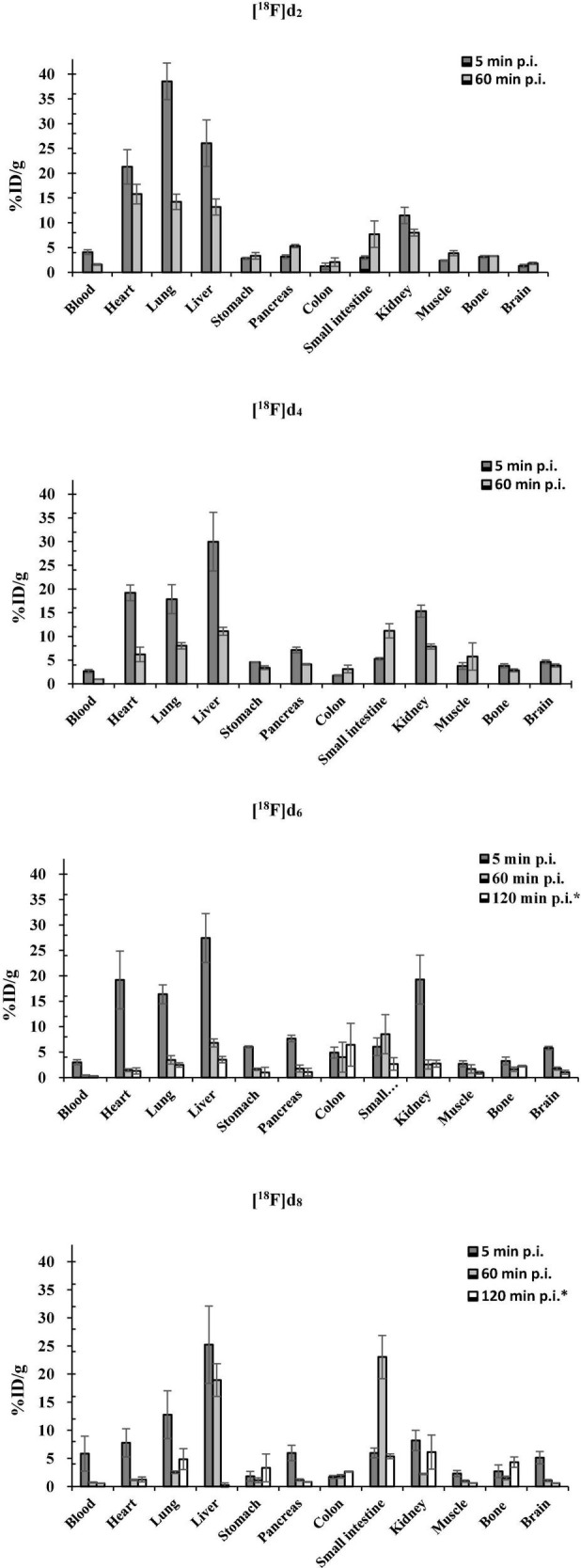
Biodistribution of [^18^F]d_2_, [^18^F]d_4_, [^18^F]d_6,_ and [^18^F]d_8_ in healthy C57BL/6J mice (*biodistribution after PET dynamic scans).

The 6-fluoropyridine ligands **d**_6_ and **d**_8_ (Figure 7 and Supplementary Material) also showed a high binding affinity to α-syn (Table 3), notwithstanding the lack of the methoxy hydrogen bond acceptor in the 3-fluoroanisole ligands. It is however more evident here the role that the heterocyclic nitrogen might have played in the increment of the propensity to forming π–π interactions (Figure 11 and Supplementary Material), as well as the reduction in hydrophobic interactions. Therefore, ligand **d**_6_ showed similar affinity as **d**_2_ to α-syn in as much as the ability of the additional hydrogen bond acceptor in the pyridine ring to form hydrogen bonds is diminished due to the fluorine atom ortho to it. In docking studies, the heterocyclic nitrogen in **d**_4_ was shown to slightly decrease the distance between His50 of α-syn and the Ar_R_ ring of the DABTAs thereby improving π–π interactions in comparison to **d**_2_ (Figure 11 and Supplementary Material). Selectivity over the other aggregating proteins (Table 3) was the highest in the least lipophilic **d**_8_, but this trend was not observed in **d**_6_. Maybe because of its lack of a good hydrogen bond contributor compared to others. In as much as **d**_4_ has a hydrogen bond acceptor on both sides, it still suffered the lack of heterocyclic nitrogen on Ar_R_, which improves π–π interactions, and steric hindrance due to the methoxy group positioned ortho to the fluorine atom which might have reduced halogen bonding. Possible steric hindrance to halogen bonding might also explain the reason why **d**_6_ has almost the same affinity to α-syn as **d**_2_, although the latter beat it out based on other metrics already mentioned which seemingly play a role in the binding properties of the DABTAs (Figure 10).

Also, based on the structures of **d**_2_, **d**_4_, **d**_6_, and **d**_8_, an in-house machine learning model was built which also showed a good correlation between the calculated (°K_i_) and experimental (K_i_) binding affinities of the DABTAs to tau and Aβ (Table 3).

There was a correlation between the already mentioned physicochemical properties (Table 2) and the brain pharmacokinetics of the ligands. With the exception **[**^18^**F]d**_6_ which showed the highest brain uptake at 5 min p.i. (Figure 11) counterintuitive to its *fp*,(Table 2) suggesting a fairly reversible binding to blood proteins. The exchange of the 3-fluoroanisole with a 6-fluoropyridine molecule led to an improvement in the brain uptake of the tracers **[**^18^**F]d**_6_ and **[**^18^**F]d**_8_ (Figure 11). Although there might be other reasons for this improvement in brain uptake, the most obvious contribution was the decrease in molar weight (MW) afforded by the above exchange (nearly 1.3 times). The number of rotatable bonds (RBN) might have contributed also contributed, with the 3-fluoroanisole tracers having one more than the 6-fluoropyridine tracers (Hou et al., 2006). Finally, in this study, it could be observed that the inclusion of a nitrogen atom in the DABTAs not only reduces their lipophilicity but also increases their brain bioavailability, as can be seen in pairs **[**^18^**F]d**_2_/**[**^18^**F]d**_4_ and **[**^18^**F]d**_2_/**[**^18^**F]d**_6_ (disregarding the differences in MW) (Figure 11). However, as was seen in **[**^18^**F]d**_6_/**[**^18^**F]d**_8_, the presence of the more basic nitrogen atom in the 2,3-methylenedioxypyridine moiety (Ar_R_) (Figure 1) in **[**^18^**F]d**_8_ led to a reduction in the brain concentration of the tracer at 5 min p.i. by 11.4% (Figure 11) in comparison to its benzodioxole counterpart (**[^18^**F]d6)(Sharom, 2003; Li-Blatter and Seelig, 2010).

**FIGURE 12:**
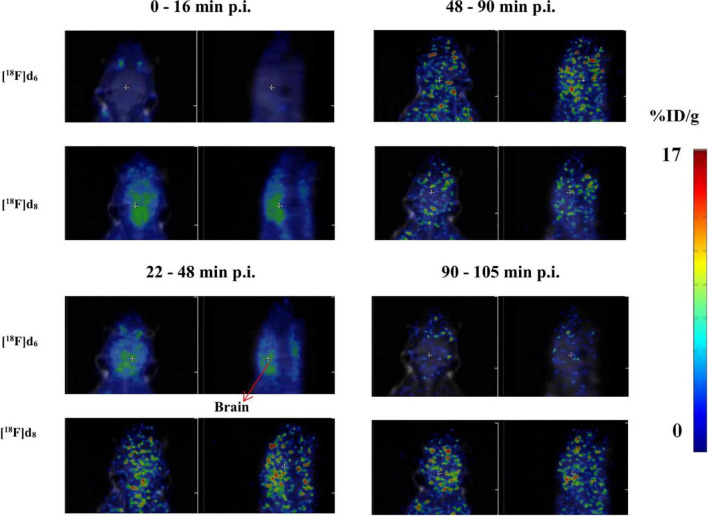
Representative axial and sagittal images of the brain accumulation of [^18^F]d_6_ and [^18^F]d_8_ displayed as SUVs.

The lowest brain uptake at 5 min p.i. was observed in the most lipophilic tracer **[**^18^**F]d**_2_. The tracer might have been retained in peripheral tissues such as the lungs or the heart as shown in Figure 11 in comparison to the other tracers (Seierstad and Agrafiotis, 2006; Raschi et al., 2009; Garrido et al., 2020). Additionally, **[**^18^**F]d**_2_ must have undergone a more extensive hepatic metabolism (Croom, 2012), which explains the increased bone concentration at 60 min p.i. Moreover, the tracer might have peaked in the brain several minutes p.i., perhaps much later than was observed for **[**^18^**F]d**_6_ in PET dynamic scans (Figures 9, 12), due to a slow release from the peripheral tissues in which it accumulated. This theory was corroborated by the 26% increase in brain content of the tracer at 60 min p.i. in comparison to that at 5 min p.i.

The brain clearance of the tracers was in accordance with their log D (Table 2). The fastest brain washout was seen in **[**^18^**F]d**_8_ (Figure 11), which was also confirmed in PET dynamic scans in healthy mice (Figure 9), after peaking between 5 and 15 min p.i. **[**^18^**F]d**_6_ peaked later between 13 and 25 min p.i., with a relatively slower washout later (Figure 9). The recorded brain uptake of **[**^18^**F]d**_6_ (Figure 9) was higher than was anticipated based on the previous biodistribution experiment carried out with the tracer (Figure 11). This might be due to a spill-over of activity mostly from the blood, since **[**^18^**F]d**_6_ showed a high plasma protein binding (PPB) (Table 2) and not due to defluorination (Figure 11); however the latter cannot be completely ruled out. Notwithstanding the relatively high brain uptake of **[**^18^**F]d**_4_, it was not further examined because its concentration in the brain at 60 min might not change significantly in an additional hour and will consequently attenuate specific signals when used in animals with the pathology.

PET images of the tracers (Figure 12) in the early time points corroborated with the *fp* (Table 2), the biodistribution (Figure 10), and the TACs (Figure 11). The tracers as expected readily underwent hepatic clearance, as reflected in their presence in the gastrointestinal system, with relatively lower renal clearance. Defluorination as seen from the images (Figure 4 and Supplementary Material), was also minimal.

The PET-MRI image of **[**^18^**F]d**_8_ from 5 to 20 min p.i. (Figure 12), showed a consistently high concentration of the tracer in the brain at this time point, hence it was decided to determine the source of the signals obtained–either from the parent tracer injected or its radiometabolites. A metabolite experiment carried out after the euthanasia of 3 mice at this time point showed that the brain content at this time point was > 99% the parent tracer **[**^18^**F]d**_8_ injected (Figure 5 and Supplementary Material). The same experiments conducted for **[**^18^**F]d**_6_ at the same time point showed the same results. Hence, although Figure 4 and Supplementary Material) showed an extensive hepatic clearance of the tracers, the products of biotransformation did not make it across the blood–brain barrier.

Examination of plasma also collected at the same time point showed that the tracers showed adequate *in vivo* stability, with more than 60% of the parent tracer remaining in the plasma in both cases (Figure 8 and Supplementary Material). Interestingly, it can be seen that **[**^18^**F]d**_6_ which showed a higher PPB, underwent relatively minimal metabolism, due to the protective effect of PPB. It was also retained longer in the blood than its less-binding counterpart **[**^18^**F]d**_8_ (Croom, 2012; Wanat, 2020).

## Conclusion

The selected tracers presented in this article all showed adequate binding affinity to the target, α-syn fibrils and excellent selectivity over Aβ and tau aggregates. These values are consistent with the performed *in silico* simulations. It can be concluded, based on these findings, that the linear scaffold of the DABTAs favors van der Waals interaction with the α-syn over ligands with a more compact structure such as the DCVJ. Using metadynamics simulations of the free energy, it was possible to identify two surface sites that are favorable for binding of the two most potent ligands **d**_4_ and **d**_8_. This information enables the fine-tuning of the tracer structures to obtain tracers with better binding affinity to α-syn.

Ruthenium complexation facilitates the radiofluorination of phenolic precursors *via* deoxyfluorination to give moderate-to-high yields and offers more room for further optimization of DABTAs as ^18^F-labeled radiotracers as well as other phenolic precursors that might be promising radiotracers for other targets.

**[**^18^**F]d**_8_ furthermore shows highly promising *in vivo* properties, that is, optimal brain uptake in mice (>4% ID/g), relatively fast brain clearance at 1-h (∼1%ID/g) and 2-h p.i. (<1% ID/g), good *in vivo* stability (>60% at 20 min p.i.), no brain radiometabolites at the same time point, and a moderately high RCY (≥25%). The postmortem human autoradiography studies with NDD brain materials are a very important part of translational research with DABTA and are currently being conducted. More experiments backed by *in silico* simulations and machine learning, in animal models with an α-synucleinopathy are in progress to further characterize the efficacy of the DABTA tracer as a non-invasive *in vivo* α-syn PET agent.

## Data Availability Statement

The original contributions presented in the study are included in the article/Supplementary Material, further inquiries can be directed to the corresponding author.

## Ethics Statement

This animal study was reviewed and approved by the Bavarian authority.

## Author Contributions

BY and BU: research project conception and chemistry. BU: radiochemistry, in vivo studies, PET, organization, execution, and analysis—design and execution. BU, BY, JL, and HÅ: manuscript preparation—writing of the first draft. JL and HÅ: in silico experiments. WP, BU, and PS: binding experiments. BU: other in vitro experiments. BU, BY, JL, HÅ, WW, WP, and PS: review and critique. All authors contributed to the article and approved the submitted version.

## Conflict of Interest

The authors declare that the research was conducted in the absence of any commercial or financial relationships that could be construed as a potential conflict of interest.

## Publisher’s Note

All claims expressed in this article are solely those of the authors and do not necessarily represent those of their affiliated organizations, or those of the publisher, the editors and the reviewers. Any product that may be evaluated in this article, or claim that may be made by its manufacturer, is not guaranteed or endorsed by the publisher.

## Footnotes

1 FDA/CDERB Available online at: https://www.accessdata.fda.gov/drugsatfda_docs/label/2014/204677s000lbl.pdf.

2 FDA/CDERA Available online at: https://www.accessdata.fda.gov/drugsatfda_docs/label/2012/202008s000lbl.pdf.

3 FDA Available online at: https://www.accessdata.fda.gov/drugsatfda_docs/label/2017/203137s008lbl.pdf.
