# Supplementary material for: Toward Novel [18F]Fluorine-Labeled Radiotracers for the Imaging of α-Synuclein Fibrils
Source: Front Aging Neurosci. 2022 Apr 29;14:830704. doi: 10.3389/fnagi.2022.830704 (PMC9099256; doi:10.3389/fnagi.2022.830704)
Supplement: Supplementary file 1 [file Data_Sheet_1.docx]

Towards Novel [^18^F]Fluorine-labeled Radiotracers for the Imaging of α-Synuclein Fibrils.

Uzuegbunam BC^1^, Li J^2^, Paslawski W^3^, Weber W^1^, Svenningsson P^3^, Ågren H^2^, Yousefi BH^5*^

^1^Department of Nuclear Medicine, Technical University of Munich, Germany. ^2^Uppsala University, Department of Physics and Astronomy, Sweden.

^3^Karolinska Institute Department of Clinical Neuroscience, Stockholm, Sweden.

^4^Department of Nuclear Medicine, Philipps University of Marburg, Germany.

**Table of Contents**

Procedure of syntheses of the DABTAs.........................................……….................................……....…….….3-9

Procedure of syntheses of the ruthenium complexes of the DABTAs.............................…….…………...…...…..9

Optimization of the radiofluorination of d_7_………………………………………………………..……..………..……10

Molar activity…………………………………………………………………………………………………………...….11

In vitro plasma stability experiments (graphs)………………..……………………………………….……………….12

Preparation of recombinant α-syn......................................................................................………...........…...….12

Preparation of the α-syn, Aβ, and tau aggregates……………………………………………………….………..13-14

Competition binding assays (graphs)……………………………………………………………….………………14-16

Metabolite experiments…………………………………………………………………………………….………...16-17

In silico studies………………………………………………………………………………………………………..17-20

References………………………………..……………………………………………………………………..…….20-21

**General methods**

All commercial reagents and solvents were used without further purification. The building blocks were purchased from Chemspace (Latvia). Semipreparative reversed-phase HPLC was performed using a Shimadzu system, which comprises 2 LC-20AP quaternary low-pressure gradient pumps, a SPD-M30A photodiode array detector, and a CBM-20A system controller. Reaction completion (it was carried out for all the synthesis carried out) and quality control for chemical synthesis and radiofluorination carried out with a Prominence analytical HPLC system (Shimadzu) with a Photo Diode Array detector (Shimadzu) and a GABI Star γ detector using a Chromolith RP18e (4.6 × 100 mm). As eluents were used water (0.1% v/v TFA) and acetonitrile (0.1% v/v TFA) with a flow rate of 3 mL/min. LC/MS experiments were carried out with LCMS-2020 ESI (Shimadzu) connected to the above analytical HPLC system. The ^1^H, and ^13^C spectra were recorded on a Bruker 500 MHz spectrometer. Sonication was performed with Ultrasonic cleaner USC-TH sonicator (VWR, Germany). In the course of experiments involving radioactivity, activity was quantified with a Capintec CRC® 15R dose calibrator. In the in vivo and ex vivo experiments, the activity in the tissue/organ samples were measured using a Perkin-Elmer Wizard 2480 automatic gamma counter. Evaporation of solvents was done with Büchi Rotavapor® R-100 and lyophilized with a Christ Alpha 1-2 LDplus freeze dryer.

1. **Chemical synthesis procedure**

**Figure 1: Step I of the chemical synthesis of the DABTAs.**

- 1. **Synthesis of the monoarylthiazole intermediates, b**

To 1.5 mL DMF solution of dithiooxamide (DTO) (180.3 mg, 1.5 mmol) in a 10 mL vial while stirring was added dropwise the corresponding α-bromo acetophenone (1 mmol): 1-(benzo[d][1,3]dioxol-5-yl)-2-bromoethan-1-one, 2-bromo-1-(3-hydroxy-4-methoxyphenyl)ethan-1-one, 2-bromo-1-(3-fluoro-4-methoxyphenyl)ethan-1-one, 2-bromo-1-(6-fluoropyridin-3-yl)ethan-1-one (2.5 mmol of DTO), 2-bromo-1-(6-bromopyridin-3-yl)ethan-1-one in 500 µL of DMF, the mixture was left to run overnight at room temperature. The reaction mixture was then centrifuged at 6000 rpm for 10 min to remove symmetrically substituted bisthiazole by-product **(c)** and the supernatant containing the product was diluted to 8 mL with 90% aqueous acetonitrile solution and purified using a semipreparative HPLC, with a Zorbax Bonus RP, 9.4 x 250 mm, 5.0 µm column. All the monoarylthiazole intermediates were obtained as greenish-yellow or yellowish-orange amorphous solids, after evaporation of the organic solvents, the samples were freeze-dried until complete dryness.

- - 1. **4-(benzo[d][1,3]dioxol-5-yl)thiazole-2-carbothioamide, b_1_ & b_6_.**

Semipreparative purification was carried out with 30% aqueous methanol solution, 0.1% THF, 0.1% TFA (solvent A) and acetonitrile, 0.1 % TFA (solvent B) with a flow-rate of 5 mL/min and a gradient A/B: 80:20 to 18:82 in 17 min. The compound was obtained in 65 - 70 % yield, with over 99% purity (HPLC). It was confirmed by ESI-MS, [M+1] = 265.0 and NMR ^1^H NMR (500 MHz, DMSO-d_6_) δ 6.07 (s, 2H), 7.00 (d, J = 5.5 Hz, 1H), 7.61 (dd, J = 4.3, 8.3 Hz, 1H), 7.69 (s, 1H), 8.27 (s, 1H), 9.94 (s, 1H), 10.19 (s, 1H). ^13^C NMR (126 MHz, DMSO-d6) δ 39.02, 39.19, 39.35, 39.52, 39.69, 39.85, 40.02, 101.30, 106.77, 108.55, 120.25, 121.60, 127.98, 147.54, 147.86, 155.27, 167.64, 186.46.

- - 1. **4-(3-hydroxy-4-methoxyphenyl)thiazole-2-carbothioamide, b_3._**

Semipreparative purification was carried out with 30% aqueous methanol solution, 0.1% THF, 0.1% TFA (solvent A) and acetonitrile, 0.1 % TFA (solvent B) with a flow-rate of 5 mL/min and a gradient A/B: 95:5 to 23:77 in 17 min. The compound was obtained in 77 % yield, with over 99% purity. It was confirmed by ESI-MS, [M+1] = 267.0 NMR ^1^H NMR (500 MHz, DMSO-d_6_) δ 3.82 (s, 3H), 7.00 (d, J = 5.6 Hz, 1H), 7.33 – 7.59 (m, 2H), 8.16 (s, 1H), 9.05 (s, 1H), 9.87 (s, 1H), 10.18 (s, 1H). ^13^C NMR (126 MHz, DMSO-d_6_) δ 55.68, 112.05, 113.71, 117.69, 121.19, 126.74, 146.57, 148.18, 155.85, 167.71, 186.62.

- - 1. **4-(3-fluoro-4-methoxyphenyl)thiazole-2-carbothioamide, b_4._**

Semipreparative purification was carried out with an aqueous mixture of THF and methanol solution (20%:10%) , 0.1% TFA (solvent A) and acetonitrile, 0.1 % TFA (solvent B) with a flowrate of 5 mL/min and a gradient A/B: 70:30 to 32:68 in 17 min. The compound was obtained in 60% yield, with over 99% purity. It was confirmed by ESI-MS, [M+1] = 269.1. ^1^H NMR (500 MHz, DMSO-d_6_) δ 3.89 (s, 3H), 7.21 – 7.29 (m, 1H), 7.85 (d, J = 8.8 Hz, 1H), 7.98 (d, J = 13.0 Hz, 1H), 8.34 (s, 1H), 9.98 (s, 1H), 10.21 (s, 1H). and NMR ^13^C NMR (126 MHz, DMSO-d_6_) δ 39.02, 39.19, 39.35, 39.52, 39.69, 39.85, 40.02, 101.30, 106.77, 108.55, 120.25, 121.60, 127.98, 147.54, 147.86, 155.27, 167.64, 186.46.

- - 1. **4-(6-bromopyridin-3-yl)thiazole-2-carbothioamide, b_7._**

Semipreparative purification was carried out with an aqueous mixture of THF and methanol solution (0.3%:40%) , 0.1% TFA (solvent A) and acetonitrile, 0.1 % TFA (solvent B) with a flow-rate of 5 mL/min and a gradient A/B: 90:10 to 20:80 in 18 min. The compound was obtained in 88% yield, with over 99% purity. It was confirmed by ESI-MS, [M+1] = 299.9 and 301.9. ^1^H NMR (500 MHz, DMSO-d_6_) δ 7.33 (d, J = 8.5 Hz, 1H), 8.54 (d, J = 3.3 Hz, 1H), 8.60 – 8.64 (m, 1H), 8.97 (s, 1H), 10.03 (s, 1H), 10.27 (s, 1H). ^13^C NMR (126 MHz, DMSO-d6) δ 39.02, 39.19, 39.35, 39.52, 39.69, 39.85, 40.02, 109.69, 109.99, 124.24, 128.16, 139.67, 139.74, 145.50, 145.63, 151.45, 161.86, 163.75, 168.62, 186.16, 186.24.

- - 1. **4-(6-fluoropyridin-3-yl)thiazole-2-carbothioamide, b_8._**

Semipreparative purification was carried out with an aqueous mixture of THF and methanol solution (0.2%:40%) , 0.1% TFA (solvent A) and acetonitrile, 0.1 % TFA (solvent B) with a flow-rate of 5 mL/min and a gradient A/B: 90:10 to 25:75 in 18 min. The compound was obtained in 42 % yield, with over 99% purity. It was confirmed by ESI-MS, [M+1] = 240.0. ^1^H NMR (500 MHz, DMSO-d6) δ 7.33 (d, J = 8.5 Hz, 1H), 8.54 (d, J = 3.1 Hz, 1H), 8.61 (d, J = 8.7 Hz, 1H), 8.97 (s, 1H), 10.03 (s, 1H), 10.27 (s, 1H). ^13^C NMR (126 MHz, DMSO-d6) δ 109.69, 124.24, 128.13, 128.16, 139.67, 139.74, 145.50, 145.63, 151.45, 161.86, 163.75, 168.62, 186.24.

- 1. **Synthesis of asymmetric DABTAs.**

**Figure 2: Step II of the chemical synthesis of the DABTAs**

- - 1. **5-(4'-(benzo[d][1,3]dioxol-5-yl)-[2,2'-bithiazol]-4-yl)-2-methoxyphenol, d_1_**

2-bromo-1-(3-hydroxy-4-methoxyphenyl)ethan-1-one (193.1 mg, 0.7879 mmol, 1.3 mol. equiv.) in 300 µL DMF was added to 160 mg (0.6060 mmol) of 4-(benzo[d][1,3]dioxol-5-yl)thiazole-2-carbothioamide dissolved in 500 µL of DMF solution in a 10 mL vial. The reaction was left to run overnight at 50°C which led to the formation of a pasty white mixture. The reaction mixture was then transferred to a 10 mL falcon tube, 5 mL of methanol was added, and the mixture was centrifuged at 6000 rpm for 10 min. The resulting sediment was then redissolved in DMSO. Both the supernatant and the redissolved sediment in DMSO solution were then purified using semipreparative HPLC. Semipreparative purification was carried out with 20% aqueous solution of THF , 0.1% TFA (solvent A) and acetonitrile, 0.1 % TFA (solvent A) with a flowrate of 5 mL/min and a gradient A/B: 40:60 to 14:86 in 18 min. The compound was obtained in ⁓80% yield, with over 98% purity as a pale yellow amorphous solid. It was confirmed by ESI-MS, [M+1] = 411.1 and NMR, ^1^H NMR (500 MHz, DMSO-*d*_6_) δ 3.82 (s, 3H), 6.09 (s, 2H), 7.03 (dd, *J* = 8.3, 10.5 Hz, 2H), 7.43 (dd, *J* = 22, 8.4 Hz, 1H), 7.48 (d, *J* = 2.2 Hz, 1H), 7.59 (d, *J* = 8.0 Hz, 2H), 8.11 (s, 1H), 8.21 (s, 1H), 9.21 (s, 1H). ^13^C NMR (126 MHz, DMSO-*d*_6_) δ 55.65, 101.37, 106.40, 108.69, 112.35, 113.48, 114.94, 115.36, 117.40, 120.30, 126.42, 127.69, 146.71, 147.58, 147.91, 148.20, 155.15, 155.64, 160.03, 160.31.

**5-(4'-([1,3]dioxolo[4,5-b]pyridin-6-yl)-[2,2'-bithiazol]-4-yl)-2-methoxyphenol, d_3_**

1-([1,3]dioxolo[4,5-b]pyridin-6-yl)-2-bromoethan-1-one (188.69 mg, 0.7731 mmol, 1.3 moL equiv.) in 500 µL DMF acidified with 25 μL of glacial acetic acid and added to 158.4 mg (0.5948 mmol) of 4-(3-hydroxy-4-methoxyphenyl)thiazole-2-carbothioamide dissolved in 500 µL of DMF acidified with 25 μL of glacial acetic acid solution in a 10 mL glass vial. The reaction was left to run overnight at room temperature, leading to the formation of a pasty brownish white reaction mixture. The reaction mixture was then transferred to a 10 mL falcon tube, 4 mL of methanol was added, and the mixture was centrifuged at 6000 rpm for 10 min. The resulting sediment was then sonicated in 10 mL of methanol till 30^o^C for 10 min twice, each time followed by centrifugation at 15^o^C to give up to 99% purity. The resulting supernatant was purified using semipreparative HPLC. Semipreparative purification was carried out with 30% aqueous methanol solution, 0.4% THF, 0.1% TFA (solvent A) and acetonitrile, 0.1 % TFA (solvent A) with a flowrate of 5 mL/min and a gradient A/B: 80:20 to 14:86 in 19 min. The product was obtained in 84.6% yield, with over 98% purity as a white amorphous solid. It was confirmed by ESI-MS, [M+1] = 412.1 and NMR, ^1^H NMR (500 MHz, DMSO-*d*_6_) δ 9.26 (s, 1H), 8.29 (d, *J* = 10.6 Hz, 2H), 8.10 (s, 1H), 7.80 (s, 1H), 7.46 (s, 1H), 7.42 (d, *J* = 7.4 Hz, 1H), 7.01 (d, *J* = 8.4 Hz, 1H), 6.21 (s, 2H), 3.81 (s, 3H). ^13^C NMR (126 MHz, DMSO-*d*_6_) δ 55.72, 100.88, 112.30, 112.40, 113.52, 115.23, 116.65, 117.52, 124.52, 126.45, 136.85, 140.54, 146.77, 148.30, 152.64, 155.75, 158.23, 159.79, 160.95.

- - 1. 4-**(benzo[d][1,3]dioxol-5-yl)-4'-(3-fluoro-4-methoxyphenyl)-2,2'-bithiazole, d_2_**

2-bromo-1-(3-fluoro-4-methoxyphenyl)ethan-1-one (14.38 mg, 0.0582 mmol, 1.2 mol. equiv.) was dissolved in 500 µL DMF and added to 20 mg (0.04849 mmol) of 4-(benzo[d][1,3]dioxol-5-yl)thiazole-2-carbothioamide dissolved in in 500 µL of DMF solution in a 10 mL vial. The reaction was left to run overnight at 45°C, after which it was transferred to a centrifuge tube. 10 mL of acetone was added to the mixture. It was then sonicated at up to 30°C for 10 min twice each time followed by centrifugation at 20^o^C at 6000 rpm to give up to 99% purity. The pure compound was dried in vacuo until all the acetone was completely evaporated to give a 90% yield of a very pale violet amorphous solid. It was confirmed by ESI-MS, [M+1] = 413.1 and NMR, ^1^H NMR (500 MHz, DMSO-*d*_6_) δ 3.90 (s, 3H), 6.09 (s, 2H), 7.04 (d, *J* = 8.0 Hz, 1H), 7.29 (t, *J* = 8.7 Hz, 1H), 7.56 – 7.61 (m, 2H), 7.80 – 7.89 (m, 2H), 8.23 (s, 1H), 8.30 (s, 1H). ^13^C NMR (126 MHz, DMSO-*d*_6_) δ 56.11, 101.37, 106.39, 108.68, 113.67, 114.13, 115.57, 116.14, 120.29, 122.65, 126.57, 127.64, 147.43, 147.91, 150.65, 152.58, 154.17, 155.20, 160.05, 160.43.

- - 1. **6-(4'-(3-fluoro-4-methoxyphenyl)-[2,2'-bithiazol]-4-yl)-[1,3]dioxolo[4,5-b]pyridine, d_4_**

1-([1,3]dioxolo[4,5-b]pyridin-6-yl)-2-bromoethan-1-one (23.65 mg, 0.09690 mmol, 1.3 mol. equiv.) in 200 µL of DMF acidified with 10 μL of glacial acetic acid and added to 20 mg (0.07454 mmol) of 4-(3-fluoro-4-methoxyphenyl)thiazole-2-carbothioamide dissolved in 300 µL of DMF solution acidified with 15 μL of glacial acetic acid in a 10 mL vial. The reaction was left to run overnight at 45°C, after which it was transferred to a centrifuge tube. 10 mL of acetone was added to the mixture. It was then sonicated till 40^o^C for 10 min twice, each time followed by centrifugation at 20^o^C at 6000 rpm for 10 min to give up to 99% purity. The pure compound was dried in vacuo until all the acetone was completely evaporated to give a 85% yield of a very pale yellow amorphous solid. It was confirmed by ESI-MS, [M+1] = 414.1. ^1^H NMR (500 MHz, DMSO-d_6_/THF-d_8_ 4:3 vol.) δ 3.92 (s, 3H), 6.22 (s, 2H), 7.25 – 7.33 (m, 1H), 7.82 – 7.89 (m, 3H), 8.29 – 8.38 (m, 3H). ^13^C NMR (126 MHz, DMSO-d_6_/THF-d_8_ 4:3 vol.) δ 55.90, 100.84, 112.00, 113.45, 113.61, 113.89, 115.99, 116.43, 122.47, 122.50, 124.52, 126.69, 126.74, 136.96, 140.48, 147.57, 147.65, 150.87, 152.80, 152.85, 154.48, 158.26, 160.27, 160.71.

- - 1. **4-(benzo[d][1,3]dioxol-5-yl)-4'-(6-bromopyridin-3-yl)-2,2'-bithiazole, d_5_**

1. bromo-1-(6-bromopyridin-3-yl)ethan-1-one (192.1 mg, 0.6886 mmol, 1.3 mol. equiv.) in 500 µL DMF was added to 140.0 mg (0.5297 mmol) of 4-(benzo[d][1,3]dioxol-5-yl)thiazole-2-carbothioamide dissolved in 500 µL of DMF solution in a 10 mL vial. The The reaction was left to run overnight at 35°C, after which it was transferred to a centrifuge tube. 10 mL of methanol was added to the mixture. It was then sonicated till 35^o^C for 10 min twice, each time followed by centrifugation at 20^o^C at 6000 rpm for 10 min to give up to 99% purity. The pure compound was dried in vacuo until all the methanol was completely evaporated to give 99% yield of an off-white amorphous solid. It was confirmed by ESI-MS, [M+1] = 444.2 and 446.2. ^1^H NMR (500 MHz, DMSO-d_6_) δ 6.10 (s, 2H), 7.05 (dd, J = 0.8, 7.7 Hz, 1H), 7.59 (d, J = 7.8 Hz, 2H), 7.80 (dd, J = 0.7, 8.3 Hz, 1H), 8.27 (s, 1H), 8.34 (dd, J = 2.6, 8.3 Hz, 1H), 8.59 (s, 1H), 9.04 (dd, J = 0.7, 2.5 Hz, 1H). ^13^C NMR (126 MHz, DMSO-d_6_) δ 101.42, 106.42, 108.74, 115.96, 119.33, 120.35, 127.58, 128.43, 128.93, 136.78, 140.96, 147.67, 147.95, 151.30, 155.31, 159.74, 161.36.
   - 1. **6-(4'-(6-bromopyridin-3-yl)-[2,2'-bithiazol]-4-yl)-[1,3]dioxolo[4,5-b]pyridine, d_7_**

1-([1,3]dioxolo[4,5-b]pyridin-6-yl)-2-bromoethan-1-one (126.8 mg, 0.4546 mmol, 1.3 mol. equiv.) in 500 µL DMF acidified with 25 μL of glacial acetic acid and added to 120 mg (0.3997 mmol) of 4-(6-bromopyridin-3-yl)thiazole-2-carbothioamide dissolved in 500 µL of DMF acidified with 25 μL of glacial acetic acid solution in a 10 mL glass vial. The reaction was left to run overnight at 45°C, after which it was transferred to a centrifuge tube. 10 mL of acetone was added to the mixture. It was then sonicated till 35^o^C for 10 min twice, each time followed by centrifugation at 20^o^C at 6000 rpm for 10 min to give up to 99% purity. The pure compound was dried in vacuo until all the acetone was completely evaporated to give 97.2 % yield of an off-white amorphous solid. It was confirmed by ESI-MS, [M+1] = 445.3 and 447.2. ^1^H NMR (500 MHz, DMSO-d_6_/THF-d_8_ 4:3 vol.) δ 6.22 (s, 2H), 7.78 (d, J = 8.8 Hz, 1H), 7.82 – 7.87 (m, 1H), 8.35 – 8.41 (m, 3H), 8.62 (s, 1H), 9.08 (s, 1H). ^13^C NMR (126 MHz, DMSO-d_6_/THF-d_8_ 4:3 vol.) δ 100.85, 111.99, 116.75, 119.17, 124.46, 128.22, 129.00, 136.51, 137.02, 140.50, 141.02, 147.88, 151.56, 153.00, 158.31, 160.34, 161.24.

- - 1. **4-(benzo[d][1,3]dioxol-5-yl)-4'-(6-fluoropyridin-3-yl)-2,2'-bithiazole, d_6_**

2-bromo-1-(6-fluoropyridin-3-yl)ethan-1-one (37.5 mg, 0.1720 mmol, 1.3 mol. equiv.) in 200 µL DMF was added to 35.0 mg (0.1324 mmol) of 4-(benzo[d][1,3]dioxol-5-yl)thiazole-2-carbothioamide dissolved in 300 µL of DMF solution in a 10 mL vial. The reaction was left to run overnight at 35°C, after which it was transferred to a centrifuge tube. 10 mL of methanol was added to the mixture. It was then sonicated till 25^o^C for 5 min twice, each time followed by centrifugation at 20^o^C to give up to 99% purity. The pure compound was dried in vacuo until all the methanol was completely evaporated to give 88.2 % yield of an off-white amorphous solid. It was confirmed by ESI-MS, [M+1] = 383.0.^1^H NMR (500 MHz, DMSO-d_6_) δ 6.07 – 6.12 (m, 2H), 7.04 (dd, J = 2.8, 7.9 Hz, 1H), 7.34 (d, J = 8.5 Hz, 1H), 7.59 (dd, J = 2.8, 10.5 Hz, 2H), 8.23 – 8.27 (m, 1H), 8.48 – 8.52 (m, 1H), 8.54 – 8.61 (m, 1H), 8.89 (s, 1H). ^13^C NMR (126 MHz, DMSO-d_6_) δ 101.40, 106.40, 108.71, 109.85, 110.15, 115.85, 118.31, 120.32, 127.59, 127.90, 127.94, 139.75, 139.81, 145.23, 145.35, 147.64, 147.93, 151.42, 155.28, 159.80, 161.20, 161.84, 163.73.

- - 1. **6-(4'-(6-fluoropyridin-3-yl)-[2,2'-bithiazol]-4-yl)-[1,3]dioxolo[4,5-b]pyridine, d_8_**

1-([1,3]dioxolo[4,5-b]pyridin-6-yl)-2-bromoethan-1-one (46.4 mg, 0.1901 mmol, 1.3 mol. equiv.) in 200 µL DMF acidified with 10 μL of glacial acetic acid and added to 35.0 mg (0.1463 mmol) of 4-(6-fluoropyridin-3-yl)thiazole-2-carbothioamide

dissolved in 300 µL of DMF acidified with 15 μL of glacial acetic acid solution in a 10 mL glass vial. The reaction was left to run overnight at room temperature, after which it was transferred to a centrifuge tube. 10 mL of 1:1 mixture of acetone and methanol was added to the mixture. It was then sonicated at 30^o^C for 5 min twice, each time followed by centrifugation at 20^o^C to give up to 99% purity. The pure compound was dried in vacuo until all the solvents was completely evaporated to give 81.9 % yield of an off-white amorphous solid. It was confirmed by ESI-MS, [M+1] = 385.3. ^1^H NMR (500 MHz, DMSO-d_6_/THF-d_8_ 4:3 vol.) δ 6.23 (s, 2H), 7.29 – 7.35 (m, 1H), 7.85 (d, J = 5.9 Hz, 1H), 8.34 – 8.40 (m, 2H), 8.54 (s, 1H), 8.61 (s, 1H), 8.93 (d, J = 3.9 Hz, 1H). ^13^C NMR (126 MHz, DMSO-d_6_/THF-d_8_ 4:3 vol.) δ 100.85, 109.59, 109.89, 112.02, 116.75, 118.24, 124.46, 127.97, 136.97, 139.55, 139.62, 140.49, 145.27, 145.40, 151.66, 152.93, 158.29, 160.42, 161.07, 162.03, 163.92.

**Synthesis of the ruthenium complexes of 5-(4'-(benzo[d][1,3]dioxol-5-yl)-[2,2'-bithiazol]-4-yl)-2-methoxyphenol, d_1_ and 5-(4'-([1,3]dioxolo[4,5-b]pyridin-6-yl)-[2,2'-bithiazol]-4-yl)-2-methoxyphenol, d_3_**

Under an atmosphere of argon, to a 4 mL vial equipped with a magnetic stirring bar, was added 120 mg of d_1_ (0.2923 mmol),(η^5^-Cyclopentadienyl)(η^6^-naphthalene)ruthenium(+)trifluoromethanesulfonate(129.6 mg, 0.2923 mmol, 1.00 equiv), acetonitrile (0.1 mL), and dichloroethane (0.9 mL). The orange suspension was heated to 60 °C. After 2 hours the suspension became a clear red solution. After 24 hours the solution was analyzed by LC-MS and roughly 80% conversion was observed. The reaction mixture was concentrated in vacuo to dryness. The brown residue was purified by HPLC on an YMC-Actus Triart C18 column ((30×150 mm, 5 μm + 30×50 mm, 5 μm), flow rate = 42.5 mL/min , 35 °C) with a linear gradient from 50:50 (0.1% TFA in H_2_O:MeOH, v:v) to 03:97 (0.1% TFA in H_2_O:MeOH, v:v) over 10 minutes. The collected fractions containing the product were combined, diluted with 50 mL brine and 20 mL distilled water. The resulting suspension was concentrated by rotary evaporation (100 mbar, 35°C) until no more methanol was evaporated. The suspension was extracted with dichloromethane (3 × 50 mL), and the combined organic layers were dried over sodium sulfate, filtered, and concentrated in vacuo to dryness to afford ruthenium phenol complex, f_1_ as a brown powder at 58 % yield). It was confirmed by ESI-MS, [M+1] = 577.2.

The synthesis of f_3_ ruthenium complex followed the same procedure with 120 mg (0.2916 mmol) of **4** and was produced as a brown powder at 56 % yield. It was confirmed by ESI-MS, [M+1] = 578.2.

1. **Radiosynthesis**.
   1. **Optimization of the radiofluorination of d_7_**

| **Table 1. Radiofluorination with Potassium carbonate (K_2_CO_3_) only.** | | | | |
| --- | --- | --- | --- | --- |
| **№** | **K_2_CO_3_, (mol. equiv. d_7_)** | **K_222_, (mol. equiv. bases)** | **T°C/Rtn duration, [min]** | **QC-HPLC yield, [%]** |
| 1. | 2.00 | 2.00 | 180/3  180/4  180/5  180/6  180/7 | 22.1  39.0  50.9  58.6  71.7 |

| **Table 2. Radiofluorination with potassium carbonate (K_2_CO_3_) and potassium oxalate ( K_2_C_2_O_4_)** | | | | | |
| --- | --- | --- | --- | --- | --- |
| **№** | **K_2_CO_3_, (mol. equiv. d_7_)** | **K_2_C_2_O_4_, (mol. equiv. d_7_)** | **K_222_, (mol. equiv. bases)** | **T°C/Rtn duration, [min]** | **QC-HPLC yield, [%]** |
| 1. | 0.01^a^ | 1.90 | 2.70 | 150/4 | 85.1 |
| 2. | 0.01 | 1.90 | 2.70 | 150/4 | 16.0 |
| 3. | 1.00 | 2.20 | 3.10 | 180/5  180/10 | 54.6/60.0  50.0 |
| 4. | 1.00 | 1.70 | 2.00 | 150/5  150/7  150/10 | 42.8  52.2  47.5 |
| 5. | 1.00 | 1.70 | 2.00 | 180/3  180/5  180/6  180/7 | 34.6  71.1/71.6  47.5  48.7/63.7 |
| 6. | 1.00 | 1.70 | 2.00 | 200/3  200/5  200/7  200/11 | 49.2/67.8  59.0/67.3  47.0/74.9  48.4 |
| **^a^ Isotopic exchange.** | | | | | |

| **Table 3. Radiofluorination with potassium carbonate (K_2_CO_3_) and potassium bicarbonate (KHCO_3_)** | | | | | |
| --- | --- | --- | --- | --- | --- |
| **№** | **K_2_CO_3_, (mol. equiv. d_7_)** | **KHCO_3_, (mol. equiv. d_7_)** | **K_222_, (mol. equiv. bases)** | **T°C/Rtn duration, [min]** | **QC-HPLC yield, [%]** |
| 1. | **-** | 2.00 | 1.50 | 140/3  140/4  140/5 | 6.6  14.0  20.8 |
| 2. | **-** | 2.00 | 1.50 | 150/3  150/4  150/5 | 10.1  28.7  26.1 |
| 3. | - ^a^ | 2.00 | 1.50 | 150/2  150/4  150/5 | 2.2  12.0  29.2 |
| 4. | **-** | 2.00 | 1.50 | 180/1  180/2  180/3 | 43.9  57.6  71.1 |
| 5. | 1.00 | 2.00 | 1.00 | 180/5  180/6  180/7  180/8  180/9 | 56.1  63.8  78.9  64.2  63.1 |
| 6. | 0.94 | 2.00 | 1.00 | 180/5  180/6  180/7  180/8  180/9 | 44.53  58.86  60.4  60.5  70.8 |
| 7. | 1.00 | 2.00 | 1.00 | 185/5  185/6  185/7  185/8  185/9 | 36.1  63.0  62.4  77.6  60.6 |
| 8. | 1.00 | 2.00 | 1.00 | 190/5  190/6  190/7  190/8  190/9 | 53.3/80.0  68.2 - 90.0  75.3/75.6  68.1/79.8  68.1/80.0 |
| 9. | 1.00 | 2.00 | 1.00 | 193/5  193/6  193/7  193/8  193/9 | 57.0  55.2  51.0  50.0  44.1 |
| 10. | 1.00^b^ | 2.00 | 1.00 | 150/5  150/7  150/9  150/11 | 2.1  2.6  4.6  4.9 |
| **^a^ Reaction in dimethylformamide (DMF)**  **^b^ Reaction in acetonitrile (ACN)** | | | | | |

K_2_C_2_O_4_: Potassium oxalate monohydrate was used, but corrected for anhydrous K_2_C_2_O_4_,

Rtn: Reaction

All reactions were conducted in dimethylsulfoxide (DMSO) unless otherwise stated.

QC-HPLC quality control of the reaction mixture at the stated temperature using the HPLC

In as much as, initial trials with the other salts gave low yields as well, it was observed that reduction of kryptofix and the use of relatively weaker basic salts, allowed to heat the reaction mixtures to higher temperatures, which resulted in the increment of the RCY.

Potassium bicarbonate (KHCO_3_) was preferred over potassium oxalate (K_2_C_2_O_4_), because the latter usually precipitated in the aqueous acetonitrile used for the elution of the [^18^F]fluoride, which led to lower elution efficiency. To keep the salt in solution, more water was usually required. This ultimately leads to lower RCY when a compensatory longer drying time does not follow, which consequently results in the loss of radioactivity via radioactive decay and as H[^18^F]F.

Solubility in aqueous acetonitrile (up to 96%) was better with KHCO_3_. Moreover, it gave higher yields at 180 °C (Table 3) in comparison to K_2_C_2_O_4_ (Table 2) both in combination with K_2_CO_3_ and when used alone. However, it could be noticed that less kryptofix was used with the former, which might mean that the d_7_ might also be sensitive to the cryptand used in the synthesis. The isolated RCY at 180 °C follows the order: KHCO_3_/K_2_CO_3_> KHCO_3_> K_2_CO_3_>K_2_C_2_O_4_/ K_2_CO_3_> K_2_C_2_O_4_in the conditions presented in Tables 1, 2 and 3.

Despite obtaining a good yield at 180 °C in a duration of 3 min with only KHCO_3_, this condition was not further used. This was because of the low elution efficiency (50% in most cases) observed when [^18^F]fluoride is eluted only with an aqueous acetonitrile solution of KHCO_3_ (pK_b_ 7.6) from the QMA cartridge. The same was seen with elution with only K_2_C_2_O_4_(pK_b_ 10), in addition to more loss of radioactivity as H[^18^F]F. One molar equivalent of K_2_CO_3_(pK_b_ 3.8) to the precursor (pK_b_ 3.8) improved the elution efficiency to over 88% in most cases.

1. **In vitro studies.**
   1. Molar activity

The molar activities of [^18^F]d_2_, [^18^F]d_4_, [^18^F]d_6_ and [^18^F]d_8_ were evaluated as follows: aliquots (100 µL) of the tracers were counted for radioactivity using a Capintec ɣ-counter. It was then corrected for decay from the end of radiosynthesis and subsequently evaluated by analytical HPLC. The HPLC UV response was measured against a calibration curve that was prepared with the cold reference standards of d_2_, d_4_, d_6_ and d_8_ to determine the concentration associated with the decay-corrected radioactivity of the injected aliquot.

- 1. In vitro plasma stability experiments.

**[^18^F]d_4_**

**[^18^F]d_2_**

_
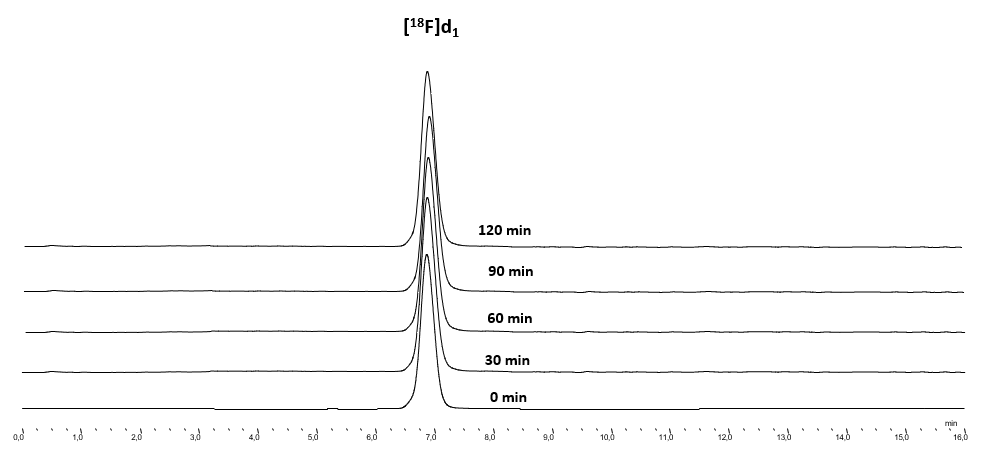

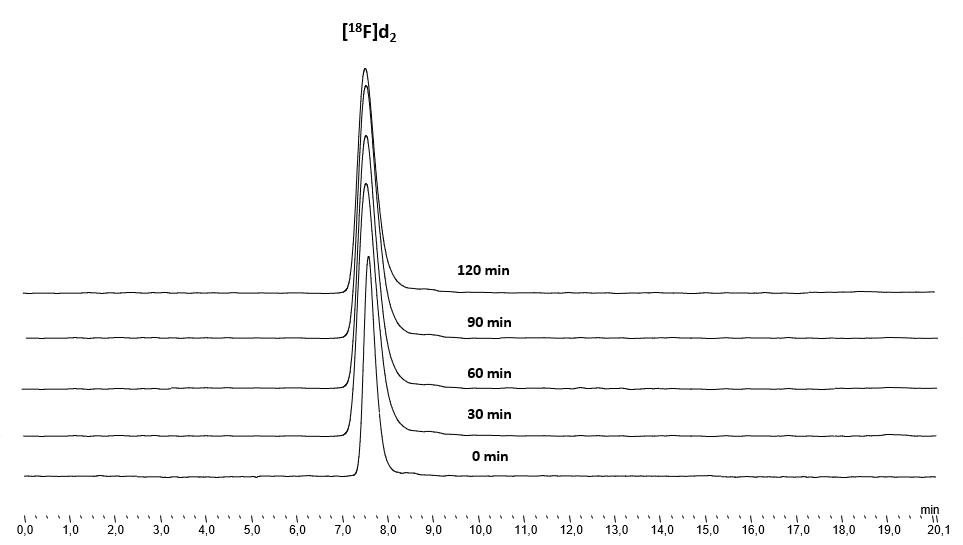
_


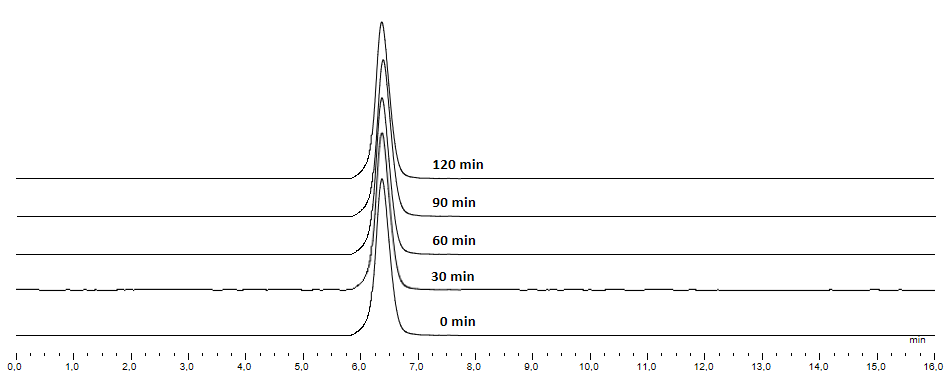

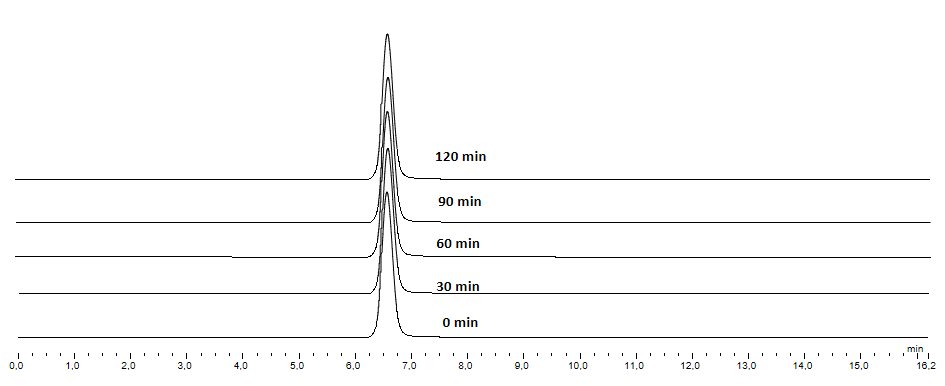


**[^18^F]d_8_**

**[^18^F]d_6_**

**Figure 3: Plasma stability chromatograms of [^18^F]d_2_, [^18^F]d_4_, [^18^F]d_6_, and [^18^F]d**_8._

**3.3. Binding assays.**

**3.3.1. Preparation of Recombinant α-syn.**

Recombinant α-syn was prepared as described previously [1,2]. Briefly, plasmid vector pET11-D, containing the insert coding human α-syn, was expressed in E.coli BL21(DE3) competent cells using an auto-induction method. Cells were harvested by centrifugation and treated with an osmotic shock buffer (20mM Tris-HCl, pH-7.2, 40% sucrose) and left in it for 10 min and centrifuged again. Afterwards, the pellet was suspended in ice-cold deionised water, with subsequent addition of saturated MgCl_2_, and briefly incubated on ice. The periplasmic fraction of the cell lysate was collected and majority of unwanted proteins precipitated by adjusting pH to 3.5 with 1M HCl. Soluble proteins were collected by centrifugation and pH of the obtained supernatant was adjusted to pH 7.5 with 1M NaOH. The solution was filtered and fractionated on a Q-Sepharose column connected to an ÄKTA Explorer system (GE Healthcare) using rising concentration of NaCl. Fractions containing α-syn were identified by SDS-PAGE and combined. Furthermore, high molecular weight aggregates were removed by filtration through a 30kDa cut-off filter, to obtain only monomeric form of α-syn, and re-analysed with SDS-PAGE to ensure purity. The final solution was dialyzed against deionised water. The α-syn concentration was determined using NanoDrop ND-1000 (Thermo Scientific), protein was aliquoted, lyophilized and stored at -20°C.

**3.3.2. Preparation of the protein aggregates.**

**3.3.2.1. Preparation of recombinant α-syn fibrils.**

Purified recombinant α-syn monomer (10 mg/mL) was incubated in PBS, pH 7.4, at 37 °C by shaking at 900 rpm in an Eppendorf Thermomixer for up to 84 hours. To determine the concentration of fibrils, the fibril reaction mixer was centrifuged at 12000 g for 5 min and the fibril pellet was washed 3 times with 20 mM Tris-HCl pH 7.4 to separate fibrils from monomer. Supernatant from all the centrifugation steps were combined and the concentration of monomer in the supernatant was determined using SDS-PAGE. The concentration of the fibrils formed was determined by subtracting the mount of α-syn monomer in the supernatant from initial amount use for fibrillation process.

**3.3.2.2. Preparation of recombinant β-amyloid fibrils.**

1 mg of β-amyloid_1−42_ peptide was dissolved in 50 μL DMSO. 925 μL of Milli-Q water was added to the above. Then 25 μL of 1M Tris HCl, pH 7.4 was added to bring the final peptide concentration to 1 mg/mL. The dissolved peptide was then incubated for 30 h at 37°C with shaking at 900 rpm. To determine the concentration of fibrils, the fibril reaction mixer was centrifuged at 12000 g for 5 min and the fibril pellet was washed three times in 20 mM Tris-HCl pH 7.4 to separate fibrils from monomer. Supernatant from all centrifugation steps were combined and the concentration of monomer in the supernatant was determined in a BCA assay, alongside a BSA standard curve. The concentration of the fibrils formed was determined by subtracting the amount of β-amyloid_1−42_ monomer in the supernatant from initial amount used for fibrillation.

**3.3.2.3. Preparation of recombinant tau fibrils.**

Recombinant tau monomer was dissolved at 300 µg/mL concentration in 20 mM Tris HCl pH 8.0, 100 mM NaCl, 25 μM low molecular weight heparin, and 0.5 mM DTT. The dissolved peptide was incubated at 37°C for 48 hours with shaking at 900 rpm. To determine the concentration of fibrils, the fibril reaction mixer was centrifuged at 12000 g for 5 min and the fibril pellet was washed 3 times in 20 mM Tris-HCl pH 7.4 to separate fibrils from monomer. Supernatants from all centrifugation steps were combined and the concentration of monomer in the supernatant was determined using BCA assay, alongside a BSA standard curve. The concentration of the fibrils formed was determined by subtracting the amount of tau monomer in the supernatant from initial amount used for fibrillation.

**3.3.2.4. Preparation of α-syn, Aβ_1−42_, and Tau Fibrils for Binding for Competition binding assays.**

The pellets of the fibrils obtained above (3.3.2.1-3) were resuspended in 20 mM Tris-HCl pH 7.4 and diluted as necessary for the binding assays.

**3.3.3. Competition binding assays**

**Figure 4. 9-(2,2-dicyanovinyl)julolidine (DCVJ)**

**d_4_**1

**d_2_**1

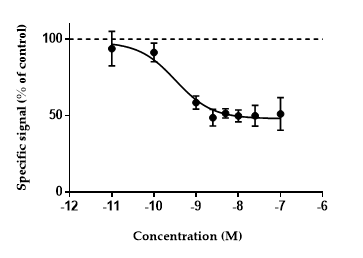


**d_8_**1

**d_6_**1

**Figure 5. α-Syn displacement binding curves of cold references of [^18^F]d_2_, [^18^F]d_4_,** **[^18^F]d_6_, and [^18^F]d_8_ against [^3^H]DCVJ**

**d_4_**

**d_2_**

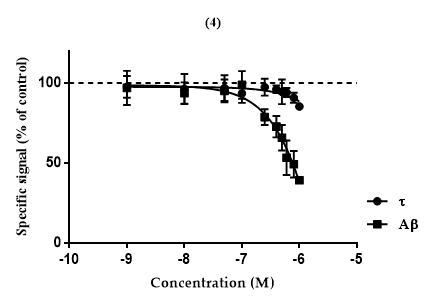


**d_8_**1

**d_6_**1

**Figure 6. Tau and Aβ displacement binding curves of cold references of [^18^F]d_2_, [^18^F]d_4_, [^18^F]d_6_, and [^18^F]d_8_ against [^3^H]DCVJ**

**4. Ex vivo, In vivo and metabolite studies.**

All animal experiments were approved by local authorities (animal license: 55.2-1-54-2532-216-15) and handled according to guidelines for the welfare and use of animals in experimental procedures.

| **[^18^F]d_6_ [^18^F]d_8_** | **[^18^F]d_6_ [^18^F]d_8_** | **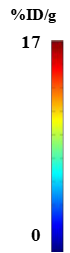**  SUV 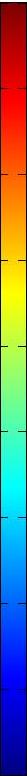 0  4  SUV 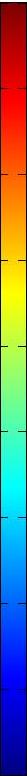 0  4 |
| --- | --- | --- |
| **0 - 16 min p.i.** | **48 - 90 min p.i.** |  |
| **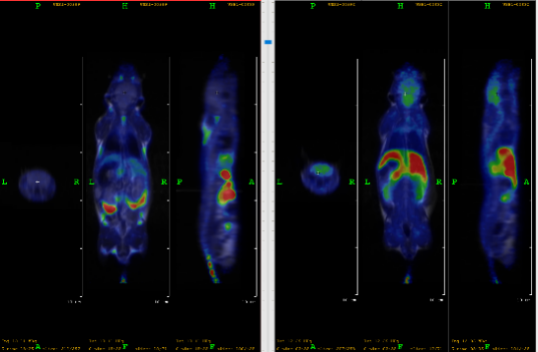 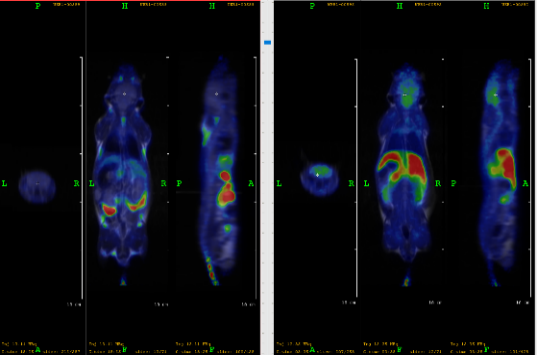** | **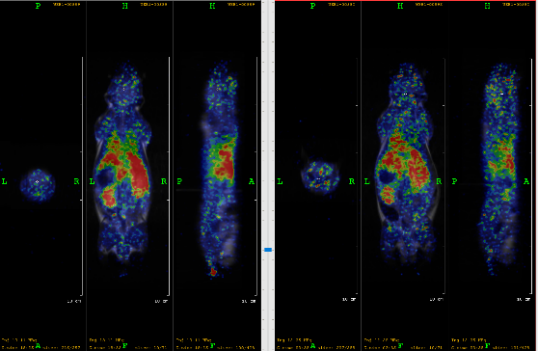 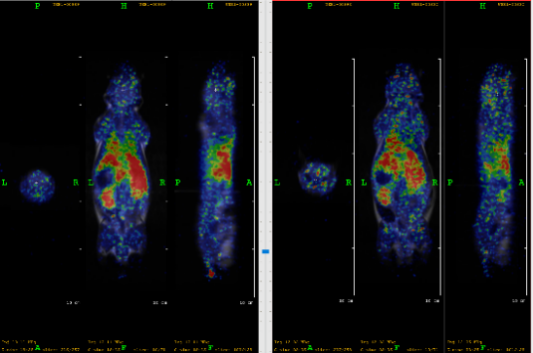** |  |
| **22 - 48 min p.i.** | **90 - 105 min p.i.** |  |
| 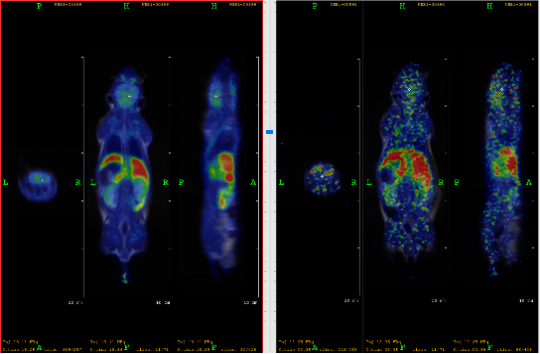 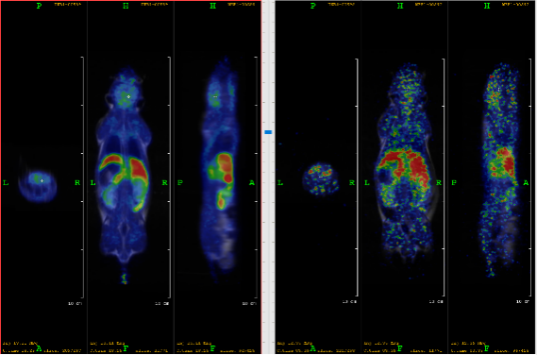 | 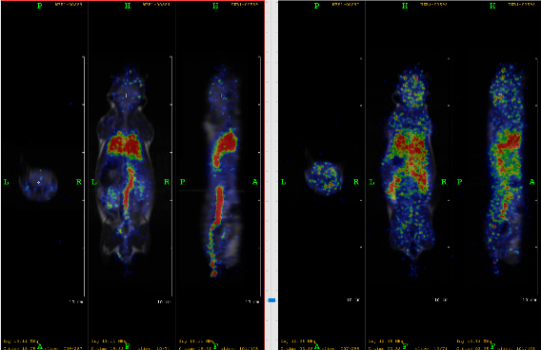 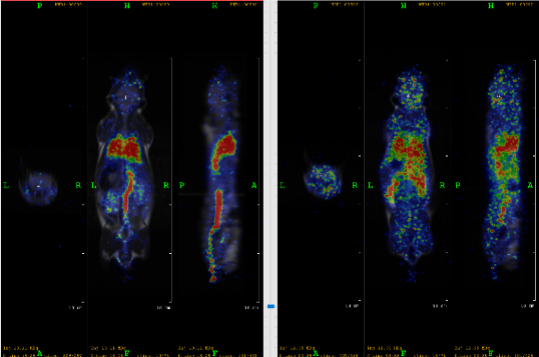 |  |

**Figure 6. Representative axial, coronal, and sagittal images of the whole-body accumulation of [^18^F]d_6_ & [^18^F]d_8_ displayed as %ID/g**

4.1. Metabolite experiments

The chromatograms were re-plotted using Origin2021.

**PT**

**PT**

**[^18^F]d_8_**

**[^18^F]d_6_**

**Figure 7. Representative HPLC chromatograms of the brain homogenates of mice injected with [^18^F]d_6_ & [^18^F]d_8_. (PT parent tracer).**

**PT**

**PT**

**[^18^F]d_8_**

**[^18^F]d_6_**

**Figure 8. Representative HPLC chromatograms of the plasma of mice injected with [^18^F]d_6_ & [^18^F]d_8_. (PT parent tracer).**

**5. In silico Studies**

**5.1. Building of the in-house machine learning model**

Machine learning has been extensively used in drug discovery and has been established as a useful tool in drug discovery. We choose the extreme gradient boosting (Xgboost) algorithm to build the regression-based machine learning model. The dataset for training was collected and curated from literature. The MACSS fingerprint was used for training the small molecules from the structures of small molecules, which was generated using the rdkit library in Python. A 10-fold cross validation was used to evaluate the quality of the model by the sklearn library in Python. We used the leave-one method to perform our predictions, in which all compounds, excluding the one to be predicted, were used for model training. We also excluded compounds with different scaffolds, that is, non- DABTA compounds) in order to avoid the introduction of noise to the regression model.

**5.2. System preparation and molecular dockings**

The prepared structure of alpha-synuclein fibril was adopted from our previous study (PDB code: 2N0A) [3,4]. The fuzzy coat was removed. The protein preparation wizard module in Schrödinger suite is used to add hydrogen atoms and determine the protonation states of ionizable residues. The initial structures of the tested compounds obtained from Chemdraw were submitted to the Ligprep module of Schrödinger suite to generate the three-dimensional coordinates and determine the protonation states. The Glide module was used for blind dockings, in which we use a normal inner and outer box sizes, 10 and 20 Å, respectively [5,6]. Multiple docking box centers were assigned through the X, Y, and Z direction of the entire fibril with the spacing interval of 1.0 Å (Figure 9). The standard precision (SP) mode was used for dockings, while other settings were left as default in Glide.

**5.3. MM/GBSA Calculations**

The docking predicted binding modes were subjected to the Prime module of Schrödinger Suite directly for the MM/GBSA calculations. MM/GBSA is an end-point free energy calculation method that is featured for fast speed and acceptable accuracy to re-score docking derived binding modes [7]. The protein-ligand complexes were refined and optimized using the OPLS3e force field with the VSGB (variable dielectric surface generalized Born) continuum solvation model [8]. For the minimization, the residues within 5 Å of the ligand were included. After that, the MM/GBSA method implemented in the Prime module was used to re-score the binding poses.

**5.4 Metadynamics Simulations**

To understand the binding profile of the DABTAs to the surface area of α-syn fibril, we have further performed well-tempered metadynamics simulations. Gromacs (version 2019.6) patched with PLUMED (version 2.6.0) was used for the simulations [9-11]. The DABTAs ligands were randomly placed in the solvent molecules so that the binding and unbinding to various surface sites of α-syn fibril can take place many times during the simulations. The protein and tracer atoms were parameterized with Amber 99SB-ildn and the general Amber force field (GAFF) [12,13]. The partial charges of ligand atoms were derived through the standard restrained electrostatic potential (RESP) fitting procedure, in which the grid points of electrostatic potential were generated from Gaussian 09 (rev. E01) at the Hartree-Fock level using the 6-31G* basis set. After the placement of TIP3P water molecules, the counter ions were added to neutralize the system and increase the ionic ionic strength to 0.15M (NaCl). The system was energy minimized, followed by the restrained equilibrations in 100-ps NVT ensemble (T=300K) and NPT ensemble (T=300K, P = 1atm). Before the production of metadynamics simulations, 100 ns unrestrained molecular dynamics simulation was carried out in NPT ensemble for each system.

The production runs of metadynamics were carried out for 2-3 μs in the NVT ensemble (T=300 K). The *x* and *y* coordinates of the center of mass of the tracer are highly related to binding and unbinding of tracers on the surface sites of α-syn. We use these coordinates as collective variables (CV) 1 and 2, respectively. Since the principal axis of the fibril has been aligned along the z-axis, the z coordinate of the ligand’s center of mass was restrained in the range from -5 to 5 Å for efficient samplings for the CVs. The initial Gaussian height and the bias factor for calculating biased potential (added every 1000 steps) were set to 0.2 kcal/mol and 6, respectively [14]. The free energy surface (FES) for each ligand was obtained reweighting on the biased potential for sampled CV space with 1000 bins assigned for each CV [15]. In this manner, we were able to identify the potential tracer binding sites on the surface of the fibril.

**5.5. Supporting Figures**

**
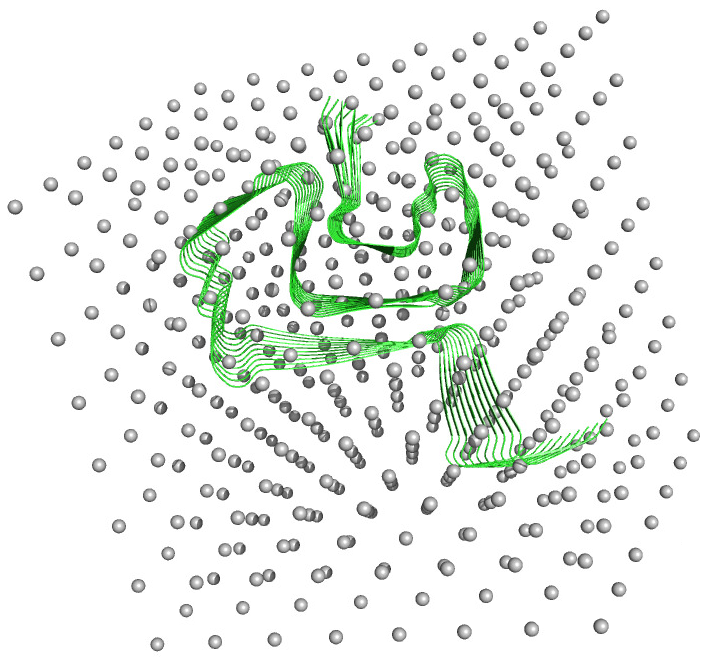
**

**Figure 9. Grid centers for the blind dockings in this work. For each grid, the inner and outer box sizes are 10, 20 Å, respectively.**


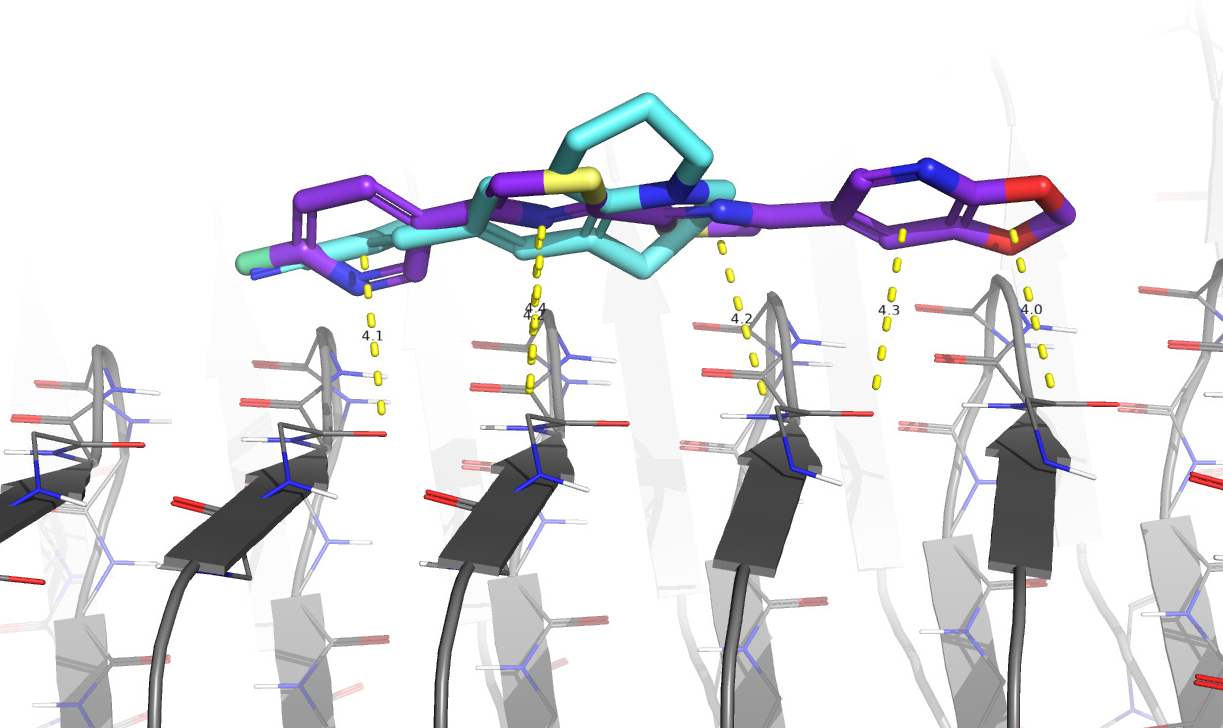


**Figure 10. Comparison of the interactions between the backbone atoms of α-syn and d_8_ (colored in marine blue) and DCVJ (colored in cyan) at Site-1**

**
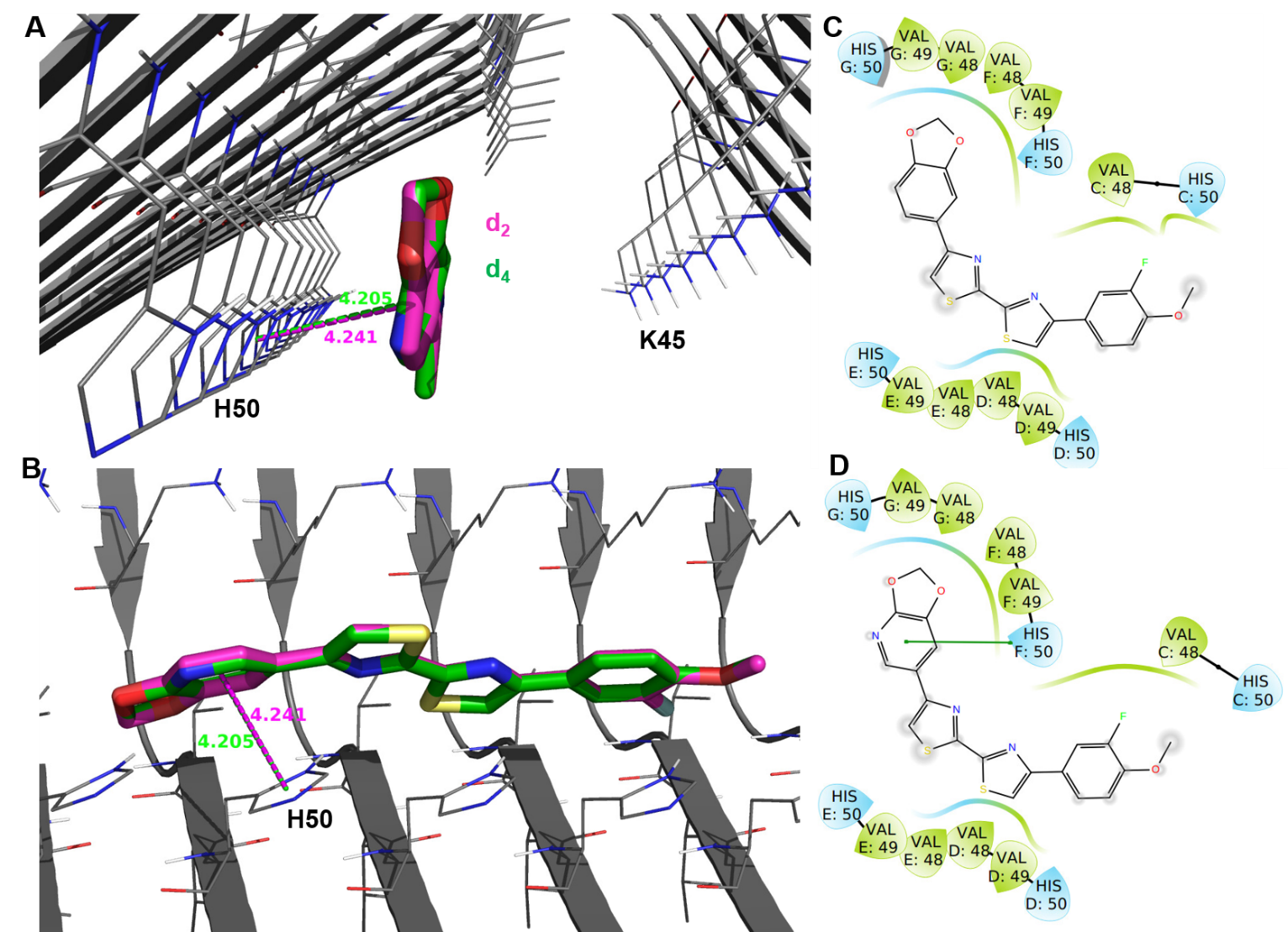
**

**Figure 11. Comparison of the binding modes of d_2_ and d_4_ at Site-4. (A) Front view of d2 and d4 at Site-4; (B) Bottom view of d2 and d4 at Site-4; (C) 2D diagram of interactions between d2 and α-syn; (D) 2D diagram of interactions between d4 and α-syn. The 2D diagram plots were generated using the “ligand interaction” module in Schrödinger Suites**

**
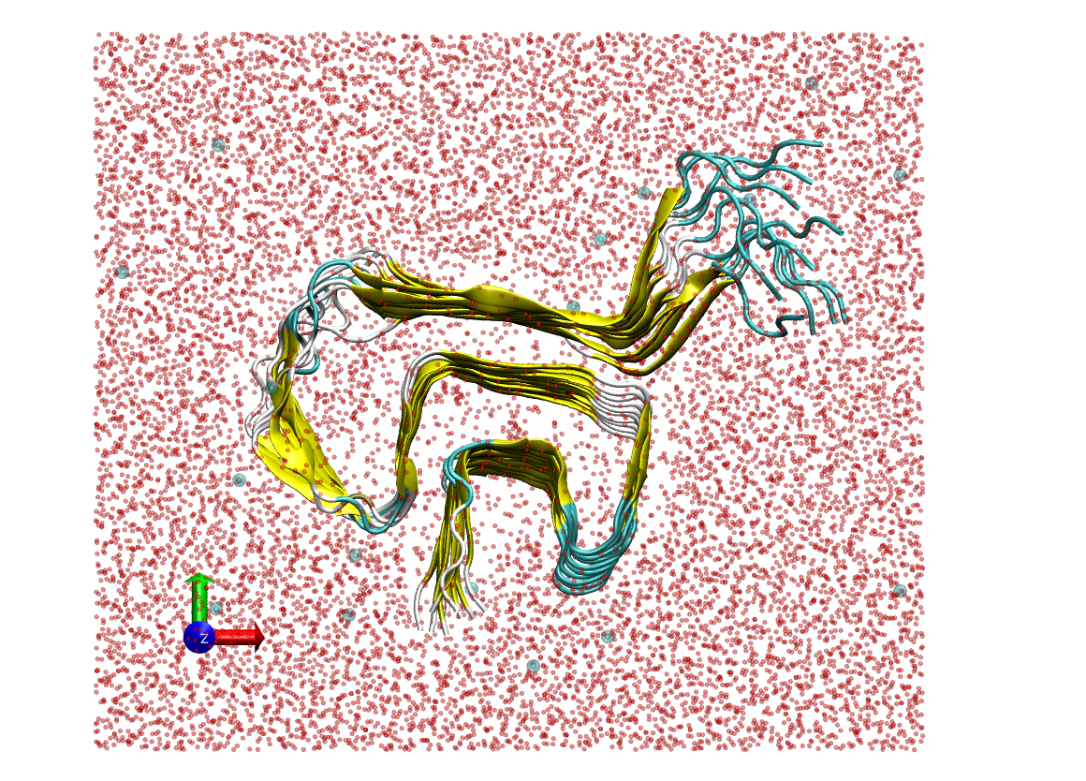
**

**Figure 11. Representation of the disassembled N-terminal area in an explicit water box during metadynamics simulations. The oxygen atoms and ions are depicted in small transparent spheres**

**References**

1. Paslawski, W.; Zareba-Paslawska, J.; Zhang, X.; Hölzl, K.; Wadensten, H.; Shariatgorji, M.; Janelidze, S.; Hansson, O.; Forsgren, L.; Andrén, P.E.; et al. α-synuclein-lipoprotein interactions and elevated ApoE level in cerebrospinal fluid from Parkinson's disease patients. *Proc. Natl. Acad. Sci. U. S. A.* **2019**, *116*, 15226–15235, doi:10.1073/pnas.1821409116.

2. Paslawski, W.; Lorenzen, N.; Otzen, D.E. Formation and Characterization of α-Synuclein Oligomers. *Methods Mol. Biol.* **2016**, *1345*, 133–150, doi:10.1007/978-1-4939-2978-8_9.

3. Kuang, G.; Murugan, A.N.; Ågren, H. Mechanistic Insight into the Binding Profile of DCVJ and α-Synuclein Fibril Revealed by Multiscale Simulations. *ACS Chem. Neurosci.* ***2019****, 10, 610–617*, doi: 10.1021/acschemneuro.8b00465.

4. Tuttle, M.D.; Comellas, G.; Nieuwkoop, A.J.; Covell, D.J.; Berthold, D.A.; Kloepper, K.D.; Courtney, M.J.; Kim, J.K.; Barclay, A.M.; kendall, A.; Wan, W.; Stubbs, G.; Schwieters, C.D.; Lee, V.M.Y.; George, J.M.; Rienstra, C.M. Solid-state NMR structure of a pathogenic fibril of full-length human α-synuclein. Nat. Struct. Mol. Biol. 2016, 23, 409-415, doi: 10.1038/nsmb.3194.

5. Friesner, R.A.; Banks, J.L.; Murphy, R.B.; Halgren, T.A.; kclictic, K.J.J.; Repasky, P.; Knoll, E.H.; Shelley, M.; Perry, J.K.; Shaw, D.E.; Francis, P.; Shenkin, P.S. Glide:  A New Approach for Rapid, Accurate Docking and Scoring. 1. Method and Assessment of Docking Accuracy. J. Med. Chem. **2004**, 47, 1739–1749, doi: 10.1021/jm0306430.

6. Halgren, T.A.; Murphy, R.B.; Friesner, R.A.; Beard, H.S.; Frye, L.L.; Pollard, T.W.; Banks, J.L. Glide:  A New Approach for Rapid, Accurate Docking and Scoring. 2. Enrichment Factors in Database Screening. J. Med. Chem. **2004**, 47, 1750–1759, doi: 10.1021/jm030644s.

7. Kollman, P.A.; Massova, I.; Reyes, C.; Kuhn, B.; Huo, S.; Chong, L.; Lee, M.; Lee, T.; Duan, Y.; Wang, W.; Donini, O.; Cieplak, P.; Srinivasan, J.; Case, D.A.; Cheatham, T.E. Calculating Structures and Free Energies of Complex Molecules:  Combining Molecular Mechanics and Continuum Models. Acc. Chem. Res. **2000**, 33, 889–897, doi: 10.1021/ar000033j.

8. Li, J.; Abel, R.; Zhu, K.; Cao, Y.; Zhao, S.; Frensner, R.A. The VSGB 2.0 model: A next generation energy model for high resolution protein structure modeling. Proteins: Struct., Funct., Genet., **2011**, 79, 2794–2812, doi: 10.1002/prot.23106.

9. Spoel, D.V.D.; Lindahl, E.; Hess, B.; Groenhof, G.; Mark, A.E.; Berendsen, H.J.C. GROMACS: Fast, flexible, and free. J. Comput. Chem., **2005**, 26, 1701–1718, doi: 10.1002/jcc.20291.

10. Branduardi, D.; Gervasio, F.L.; Parrinello, M. From *A* to *B* in Free Energy Space. J. Chem. Phys., **2007**, 126, 054103, doi: 10.1063/1.2432340.

11. Tribello, G.A.; Bonomi, M.; Branduardi, D.; Camilloni, C.; Bussi, G.; PLUMED 2: New feathers for an old bird. Comput. Phys. Commun. **2014**, 185, 604–613, doi: 10.1016/j.cpc.2013.09.018.

12. Larsen, K.L.; Piana, S.; Palmo, K.; Maragakis, P.; Klepeis, J.L.; Dror, R.O.; Shaw, D.E. Improved Side-chain Torsion Potentials for the Amber ff99SB Protein Force Field. Proteins: Struct., Funct., Genet., **2010**, 78, 1950−1958, doi: 10.1002/prot.22711.

13. Wang, J.; Wolf, R. M.; Caldwell, J. W.; Kollman, P. A.; Case, D.A. Development and Testing of A General Amber Force Field. J. Comput. Chem. **2004**, 25, 1157−1174, doi: 10.1002/jcc.20035.

14. Valsson, O.; Tiwary, P.; Parrinello, M. Enhancing Important Fluctuations: Rare Events and Metadynamics from a Conceptual Viewpoint. Annu. Rev. Phys. Chem. **2016**, 67, 159−184, doi: 10.1146/annurev-physchem-040215-112229.

15. Tiwary, P.; Parrinello, M. A Time-Independent Free Energy Estimator for Metadynamics. J. Phys. Chem. B, **2015**, 119, 736−742, doi: 10.1021/jp504920s.
